# ABCB1 as predominant resistance mechanism in cells with acquired SNS-032 resistance

**DOI:** 10.18632/oncotarget.11160

**Published:** 2016-08-09

**Authors:** Nadine Löschmann, Martin Michaelis, Florian Rothweiler, Yvonne Voges, Barbora Balónová, Barry A. Blight, Jindrich Cinatl

**Affiliations:** ^1^ Institut für Medizinische Virologie, Klinikum der Goethe-Universität, 60596 Frankfurt am Main, Germany; ^2^ Centre for Molecular Processing and School of Biosciences, University of Kent, Canterbury, UK; ^3^ School of Physical Sciences, University of Kent, Canterbury, UK

**Keywords:** ABCB1, CDK inhibitor, multi-drug resistance, neuroblastoma, cancer

## Abstract

The CDK inhibitor SNS-032 had previously exerted promising anti-neuroblastoma activity via CDK7 and 9 inhibition. ABCB1 expression was identified as major determinant of SNS-032 resistance. Here, we investigated the role of ABCB1 in acquired SNS-032 resistance. In contrast to ABCB1-expressing UKF-NB-3 sub-lines resistant to other ABCB1 substrates, SNS-032-adapted UKF-NB-3 (UKF-NB-3^r^SNS- 032^300nM^) cells remained sensitive to the non-ABCB1 substrate cisplatin and were completely re-sensitized to cytotoxic ABCB1 substrates by ABCB1 inhibition. Moreover, UKF-NB-3rSNS-032^300nM^ cells remained similarly sensitive to CDK7 and 9 inhibition as UKF-NB-3 cells. In contrast, SHEPrSNS-032^2000nM^, the SNS-032-resistant sub-line of the neuroblastoma cell line SHEP, displayed low level SNS-032 resistance also when ABCB1 was inhibited. This discrepancy may be explained by the higher SNS-032 concentrations that were used to establish SHEPrSNS-032^2000nM^ cells, since SHEP cells intrinsically express ABCB1 and are less sensitive to SNS-032 (IC_50_ 912 nM) than UKF-NB-3 cells (IC_50_ 153 nM). In conclusion, we show that ABCB1 expression represents the primary (sometimes exclusive) resistance mechanism in neuroblastoma cells with acquired resistance to SNS-032. Thus, ABCB1 inhibitors may increase the SNS-032 efficacy in ABCB1-expressing cells and prolong or avoid resistance formation.

## INTRODUCTION

Neuroblastoma is the most frequent solid extracranial pediatric cancer. About half of the patients are diagnosed with high-risk disease associated with overall survival rates below 50% despite myeloablative therapy and differentiation therapy using retinoids [1, 2].

SNS-032 (BMS-387032) is a cyclin-dependent kinase 2 (CDK2), 7, and 9 inhibitor under pre-clinical and clinical investigation for a wide range of solid and hematologic malignancies [3–16] including neuroblastoma [17–19]. The compound was initially introduced as N-acyl-2-aminothiazole inhibitor of CDK2 [3]. The relevance of CDK2, CDK7, and/ or CDK9 as crucial SNS-032 drug targets differs between different cancer cell types [4, 7–9, 12, 19]. We recently showed that therapeutic SNS-032 concentrations exerted anti-neuroblastoma effects in a panel of 109 neuroblastoma cell lines (19 parental neuroblastoma cell lines, 90 sub-lines with acquired resistance to 14 different anti-cancer drugs) and in primary neuroblastoma cells [19]. SNS-032 further inhibited tumor growth in a chemoresistant neuroblastoma xenograft model [19]. Interference with CDK7 and CDK9 appeared to be critical for the anti-neuroblastoma effects of SNS-032 [19].

SNS-032 had further been speculated to interfere with ABCB1 (also known as P-glycoprotein or MDR1) [20]. Our report (published on the 1^st^ December 2013) [19] and another report (published on 23^rd^ December 2013) [21] confirmed this assumption. ABCB1 expression was the dominant SNS-032-resistance mechanism in neuroblastoma cells from a panel of 109 neuroblastoma cell lines [19]. Only ABCB1-expressing neuroblastoma cell lines were insensitive to therapeutically achievable SNS-032 concentrations. In the presence of ABCB1 inhibitors, all 30 ABCB1-expressing neuroblastoma cell lines displayed SNS-032 IC_50_ values in the range of therapeutic SNS-032 concentrations [19].

Here, we established and characterized SNS-032-resistant sub-lines of the MYCN-amplified, ABCB1-negative neuroblastoma cell line UKF-NB-3 (UKF-NB-3^r^SNS-032^300nM^) and the non-MYCN-amplified, ABCB1-expressing neuroblastoma cell line SHEP (SHEP^r^SNS-032^2000nM^) to analyze the role of ABCB1 in neuroblastoma models of acquired SNS-032 resistance.

## RESULTS

### Enhanced ABCB1 expression in the SNS-032-resistant UKF-NB-3 sub-line UKF-NB-3^r^SNS-032^300nM^

**Figure 1:**
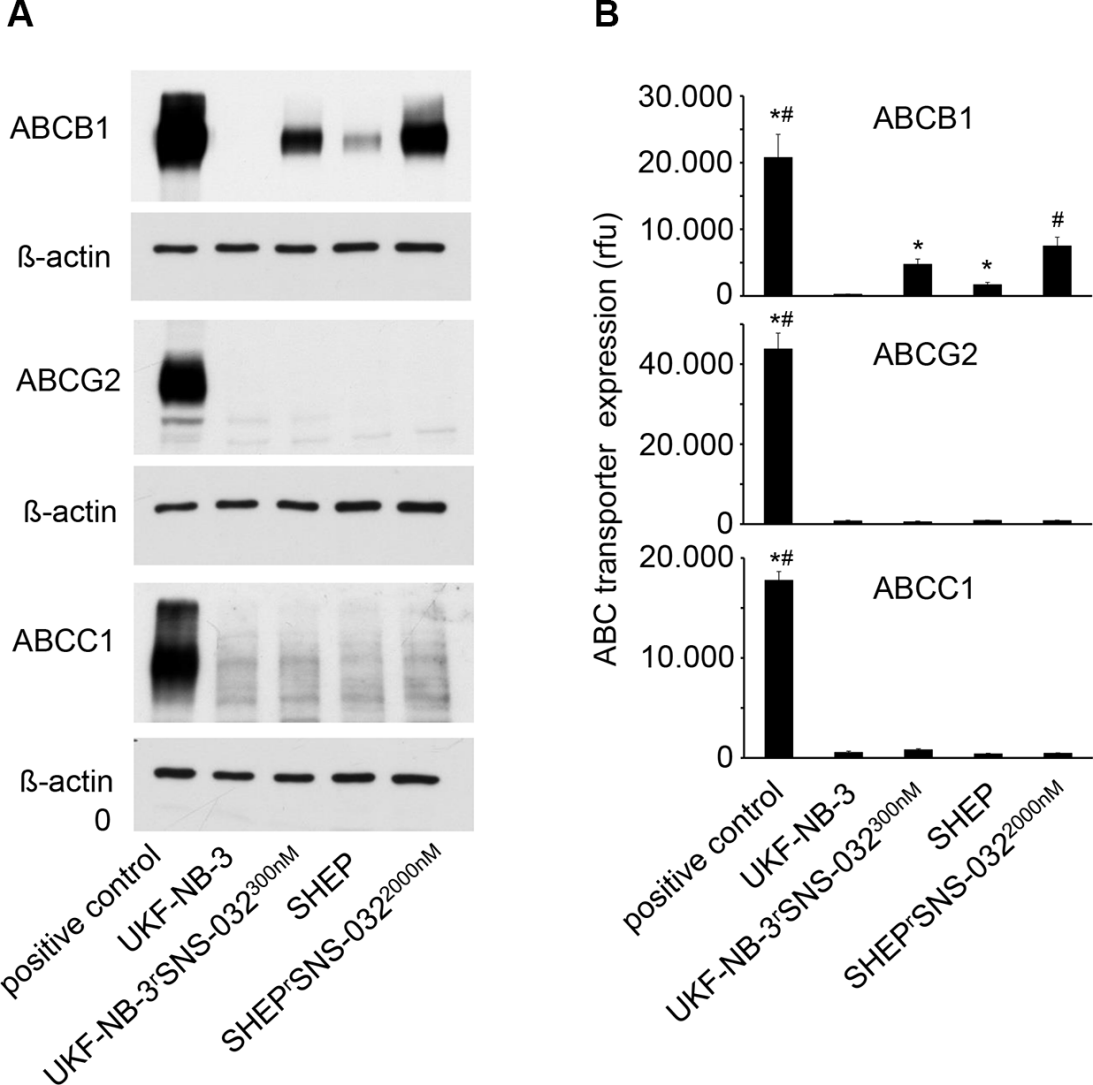
ABC transporter expression levels in in the neuroblastoma cell lines UKF-NB-3 and SHEP and their sub-lines with acquired resistance to SNS-032 resistance (UKF-NB-3^r^SNS-032^300nM^, SHEP^r^SNS-032^2000nM^) (**A**) Cropped representative Western blots indicating ABCB1, ABCG2, and ABCC1 levels. (**B**) ABCB1, ABCG2, and ABCC1 levels detected by flow cytometry and presented as relative fluorescence units (rfu). Representative flow cytometry histograms are presented in Supplementary Figure S2. * *P* < 0.05 relative to UKF-NB-3 cells, ^#^ *P* < 0.05 relative to SHEP. Positive controls were ABCB1-transduced UKF-NB-3 cells for ABCB1, ABCG2-transduced UKF-NB-3 cells for ABCG2, and NLF^r^VCR^10^ cells for ABCC1.

UKF-NB-3 cells were adapted to growth in the presence of SNS-032 300 nM by step-wise increase of the SNS-032 concentration. No pre-existing resistant sub-population could be selected by directly applying SNS-032 300 nM. The SNS-032 IC_50_ value was four times higher in SNS-032-adapted UKF-NB-3^r^SNS-032^300nM^ cells (606.7 nM) than in UKF-NB-3 cells (152.6 nM). UKF-NB-3 and its SNS-032-resistant sub-line UKF-NB-3^r^SNS-032^300nM^ were characterized by similar doubling times (UKF-NB-3: 26.6 ± 8.2 h, UKF-NB-3^r^SNS-032^300nM^ 32.9 ± 12.8 h) and a similar morphology (Supplementary Figure S1). However, UKF-NB-3^r^SNS-032^300nM^ cells displayed elevated ABCB1 levels relative to UKF-NB-3 cells (Figure 1, Supplementary Figure S2). We had previously shown that SNS-032 is also a substrate of ABCG2 (also known as BCRP) [19]. In contrast, expression of ABCC1 (also known as MRP1), another relevant ABC transporter known to be involved in cancer cell drug resistance in various cancer entities including neuroblastoma [22], did not influence anti-cancer activity of SNS-032 (Supplementary Figure S3). We did neither detect increased ABCG2 nor ABCC1 expression in UKF-NB-3^r^SNS-032^300nM^ cells (Figure 1, Supplementary Figure S2).

### Sensitization of ABCB1-expressing drug-resistant UKF-NB-3 sub-lines to SNS-032 and other ABCB1 substrates by inhibition of ABCB1

**Figure 2:**
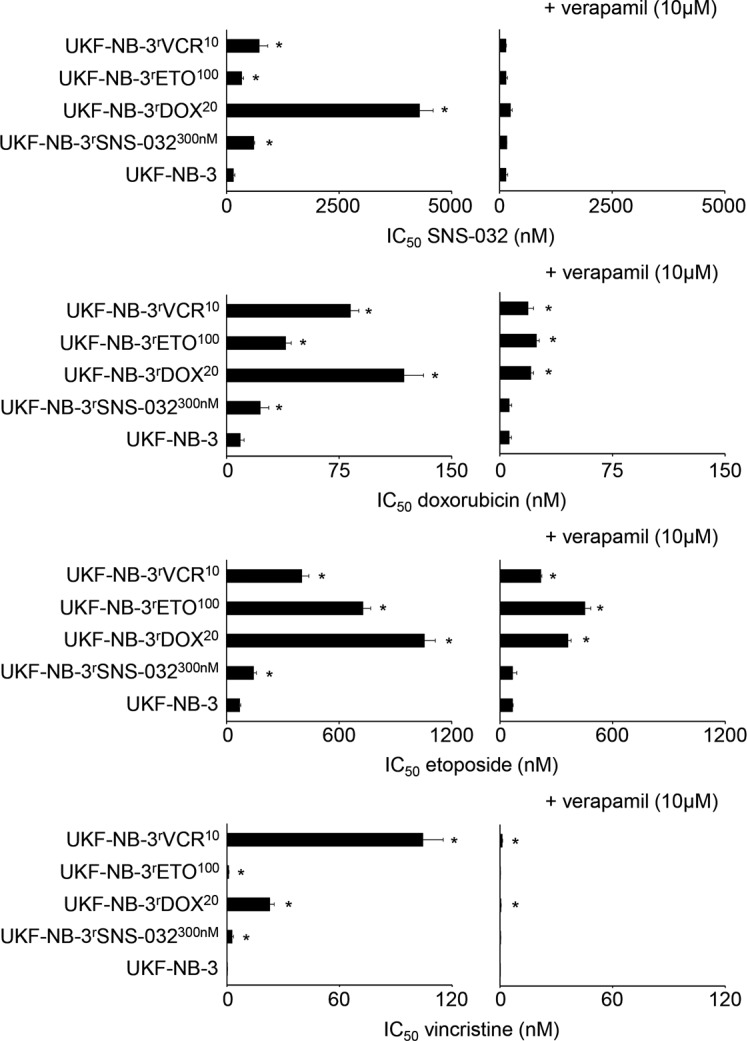
Sensitivity of UKF-NB-3 and its ABCB1-expressing sub-lines with acquired resistance to SNS-032 (UKF-NB-3^r^SNS-032^300nM^), doxorubicin (UKF-NB-3^r^DOX^20^), etoposide (UKF-NB-3^r^ETO^100^), and vincristine (UKF-NB-3^r^VCR^10^) to the cytotoxic ABCB1 substrates SNS-032, doxorubicin, etoposide, and vincristine in the absence or presence of the ABCB1 inhibitor verapamil Verapamil alone did not influence cell viability (Supplementary Table S1A). * *P* < 0.05 relative to the drug concentration that reduces cell viability by 50% (IC_50_) in UKF-NB-3 cells

**Figure 3:**
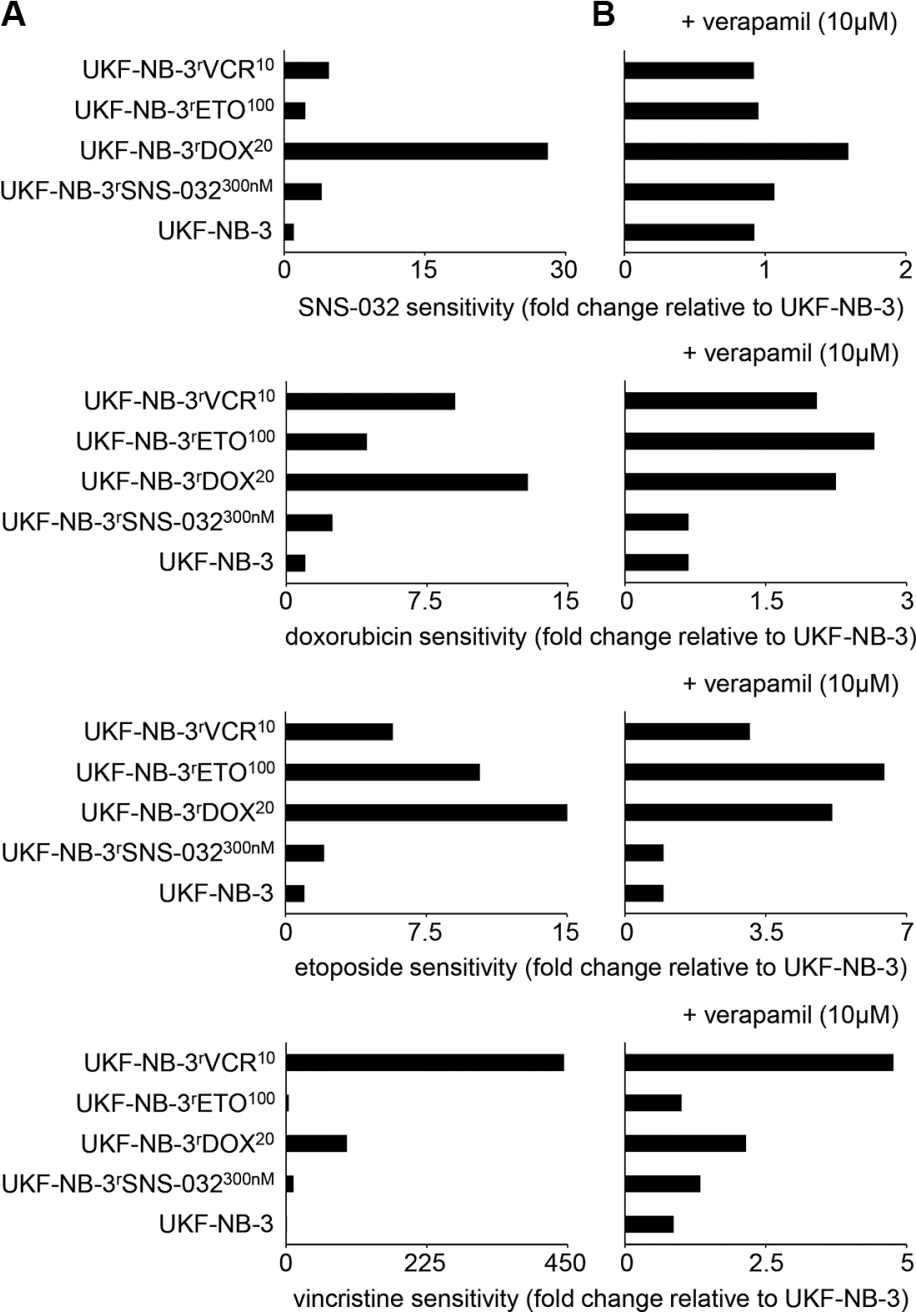
Relative sensitivity of UKF-NB-3 and its ABCB1-expressing sub-lines with acquired resistance to SNS-032 (UKF-NB-3^r^SNS-032^300nM^), doxorubicin (UKF-NB-3^r^DOX^20^), etoposide (UKF-NB-3^r^ETO^100^), and vincristine (UKF-NB-3^r^VCR^10^) to the cytotoxic ABCB1 substrates SNS-032, doxorubicin, etoposide, and vincristine in the absence or presence of the ABCB1 inhibitor verapamil (**A**) Fold change IC_50_ investigated cell line/ IC_50_ UKF-NB-3; (**B**) Fold change IC_50_ investigated cell line in the presence of verapamil (10 μM)/ IC_50_ UKF-NB-3

UKF-NB-3^r^SNS-032^300nM^ cells displayed cross-resistance to the cytotoxic ABCB1 substrates doxorubicin, etoposide, and vincristine (Figure 2, Supplementary Table S1A). The fold changes IC_50_ resistant UKF-NB-3^r^SNS-032^300nM^ / IC_50_ UKF-NB-3 ranged between 2.0 (etoposide) and 10.8 (vincristine) (Figure 3, Supplementary Table S1A). Addition of verapamil 10 μM, a concentration that did not affect the viability of the investigated cell lines (Supplementary Table S1A), re-sensitized UKF-NB-3^r^SNS-032^300nM^ to SNS-032 to the level of the parental UKF-NB-3 cells as indicated by a fold change IC_50_ SNS-032 in UKF-NB-3^r^SNS-032^300nM^ cells in the presence of verapamil/ IC_50_ SNS-032 in UKF-NB-3 cells below 2 (Figure 3, Supplementary Table S1A). Verapamil also reduced the doxorubicin, etoposide, and vincristine IC_50_ values in UKF-NB-3^r^SNS-032^300nM^ cells to a level similar to UKF-NB-3 (Figure 3; Supplementary Table S1A).

To further confirm the role of ABCB1 in UKF-NB-3^r^SNS-032^300nM^ cells, we depleted ABCB1 using siRNA. ABCB1 depletion increased SNS-032 sensitivity in UKF-NB-3^r^SNS-032^300nM^ cells. Since no complete suppression of ABCB1 expression was achieved by siRNA, the SNS-032 IC_50_ remained higher than in parental UKF-NB-3 cells (Supplementary Table S1B; Supplementary Figure S4). However, the SNS-032 IC_50_ value could be reduced in UKF-NB-3^r^SNS-032^300nM^ cells to the level of UKF-NB-3 cells by the use of zosuquidar (Supplementary Table S1C), an alternative ABCB1 inhibitor that structurally differs from verapamil [23].

Moreover, we synthesized a fluorescent SNS-032-BODIPY derivative. Flow cytometry experiments indicated, compared to UKF-NB-3, a reduced accumulation of SNS-032-BODIPY in ABCB1-transduced UKF-NB-3 (UKF-NB-3^ABCB1^) cells and UKF-NB-3^r^SNS-032^300nM^ cells that could be restored by the use of verapamil (Supplementary Figure S5). Notably, the differences between SNS-032-BODIPY accumulation in UKF-NB-3^r^SNS-032^300nM^ cells in the absence or presence of verapamil seemed to be small compared to the differences observed in UKF-NB-3^ABCB1^ cells. However, this appears to reflect the respective discrepancies in the SNS-032 IC_50_ values (UKF-NB-3^r^SNS-032^300nM^: 607 nM; UKF-NB-3^ABCB1^: 3885 nM).

The doxorubicin-resistant (UKF-NB-3^r^DOX^20^), etoposide-resistant (UKF-NB-3^r^ETO^100^), and vincristine-resistant (UKF-NB-3^r^VCR^10^) UKF-NB-3 sub-lines that express ABCB1 displayed cross-resistance to SNS-032, doxorubicin, etoposide, and vincristine. Verapamil decreased the SNS-032 IC_50_ values in all three cell lines to a level similar to UKF-NB-3 as indicated by fold changes (SNS-032 IC_50_ in resistant cell lines in the presence of verapamil/ SNS-032 IC_50_ in UKF-NB-3 cells) below 2 (Figure 3, Supplementary Table S1A). However, verapamil did not re-sensitize UKF-NB-3^r^DOX^20^, UKF-NB-3^r^ETO^100^, or UKF-NB-3^r^VCR^10^ cells to doxorubicin, etoposide, or vincristine to the level of UKF-NB-3 cells (Figure 3, Supplementary Table S1A). The only exemption was the vincristine sensitivity of UKF-NB-3^r^ETO^100^ cells (Figure 3, Supplementary Table S1A).

### Cross-resistance of ABCB1-expressing drug-resistant UKF-NB-3 sub-lines to the non-ABCB1 substrate cisplatin and of the cisplatin-resistant UKF-NB-3 sub-line UKF-NB-3^r^CDDP^1000^ to ABCB1 substrates

**Figure 4:**
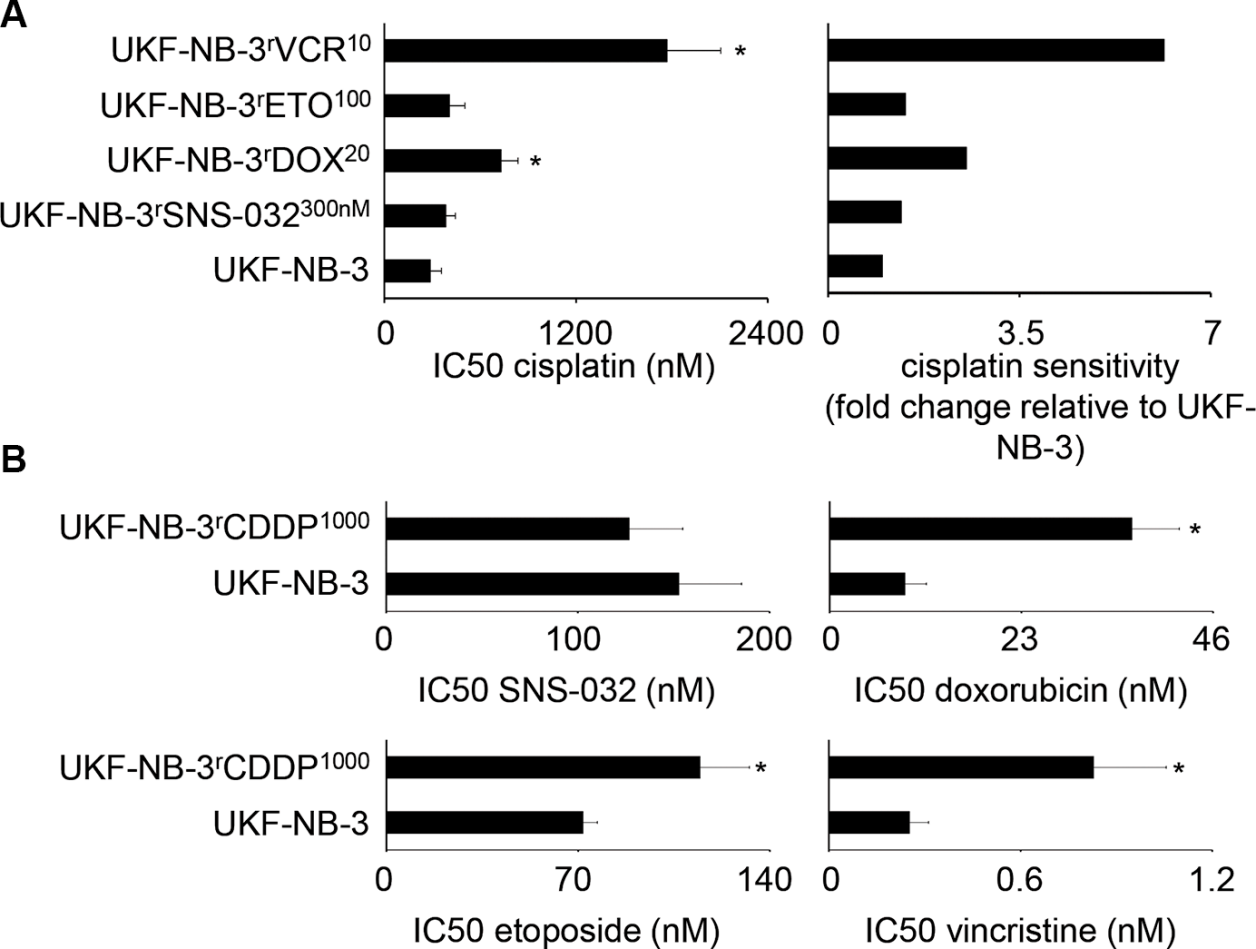
Sensitivity of UKF-NB-3 and its ABCB1-expressing sub-lines with acquired resistance to SNS-032 (UKF-NB-3^r^SNS-032^300nM^), doxorubicin (UKF-NB-3^r^DOX^20^), etoposide (UKF-NB-3^r^ETO^100^), and vincristine (UKF-NB-3^r^VCR^10^) to the non-ABCB1 substrate cisplatin, and sensitivity of the non ABCB1-expressing cisplatin-resistant UKF-NB-3 sub-line UKF-NB-3^r^CDDP^1000^ to the ABCB1 substrates SNS-032, doxorubicin, etoposide, and vincristine (**A**) Cisplatin concentrations that reduce cell viability by 50% (IC_50_) and relative cisplatin sensitivity in drug-resistant UKF-NB-3 sub-lines relative to UKF-NB-3, numerical values are presented in Supplementary Table S1D. *P < 0.05 relative to UKF-NB-3; (**B**) IC_50_ values for SNS-032, doxorubicin, etoposide, or vincristine in UKF-NB-3 or UKF-NB-3^r^CDDP^1000^ cells, numerical values are presented in Supplementary Table S1E. *P < 0.05 relative to UKF-NB-3

We next determined the resistance profile to cisplatin that is not an ABCB1 substrate. UKF-NB-3^r^SNS-032^300nM^ and UKF-NB-3^r^ETO^100^ did not display cisplatin resistance (cisplatin IC_50_ resistant UKF-NB-3 sub-line/ cisplatin IC_50_ UKF-NB-3 < 2). In contrast, UKF-NB-3^r^DOX^20^ and UKF-NB-3^r^VCR^10^ cells were substantially less sensitive to cisplatin than UKF-NB-3 cells (Figure 4A; Supplementary Table S1D).

UKF-NB-3^r^CDDP^1000^ cells displayed profound cisplatin resistance (cisplatin IC_50_: UKF-NB-3, 280 nM; UKF-NB-3^r^CDDP^1000^, 8936 nM, Supplementary Table S1D) and cross-resistance (fold change IC_50_ UKF-NB-3^r^CDDP^1000^/ IC_50_ UKF-NB-3 ≥ 2) to doxorubicin (fold change 4.0) and vincristine (fold change 3.3) but not to SNS-032 (fold change 0.8) or etoposide (fold change 1.6) (Figure 4B, Supplementary Table S1E).

### CDK7 and CDK9 as drug targets in UKF-NB-3^r^SNS-032^300nM^ cells

We had previously shown that SNS-032 reduces the viability of UKF-NB-3 cells via interference with CDK7 and CDK9 and subsequent RNA polymerase II inhibition resulting in depletion of anti-apoptotic proteins with a high turnover rate including Mcl-1, XIAP, and survivin [19].

**Figure 5:**
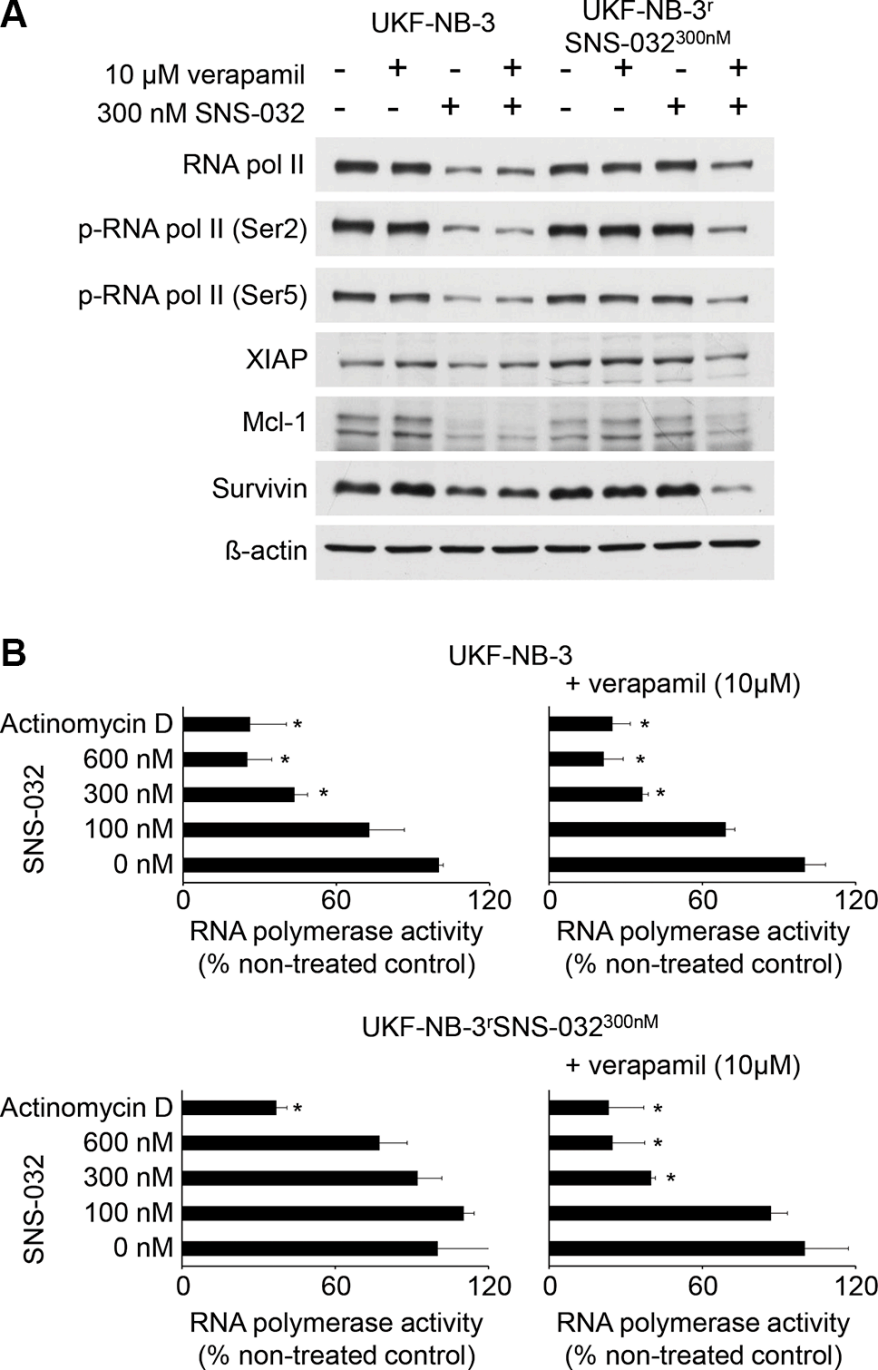
Effects of SNS-032 on CDK7 and CDK9 signalling and RNA polymerase activity in UKF-NB-3 and UKF-NB-3^r^SNS-032^300nM^ cells in the absence or presence of the ABCB1 inhibitor verapamil (**A**) Cropped Western blots indicating RNA polymerase II protein levels, RNA polymerase II phosphorylation at Ser-5 (target of CDK7), RNA polymerase II phosphorylation at Ser-2 (target of CDK9), and levels of anti-apoptotic proteins with a high rate (XIAP, Mcl-1, survivin) in UKF-NB-3 and UKF-NB-3^r^SNS-032^300nM^ cells after 24 h of incubation. β-actin served as loading control. (**B**) Effects of SNS-032 on the RNA polymerase activity in UKF-NB-3 and UKF-NB-3^r^SNS-032^300nM^ cells in the absence or presence of the ABCB1 inhibitor verapamil after 6 h of incubation. Actinomycin D 100 ng/mL served as positive control. *P < 0.05 relative to non-treated control

In the presence of verapamil 10 μM, SNS-032 300 nM exerted similar effects on RNA polymerase II protein levels, RNA polymerase II phosphorylation at Ser-5 (target of CDK7), RNA polymerase II phosphorylation at Ser-2 (target of CDK9) (Figure 5A), RNA polymerase II activity (Figure 5B), and XIAP, Mcl-1, and survivin protein levels (Figure 5A) in UKF-NB-3^r^SNS-032^300nM^ cells as in UKF-NB-3 cells.

**Figure 6:**
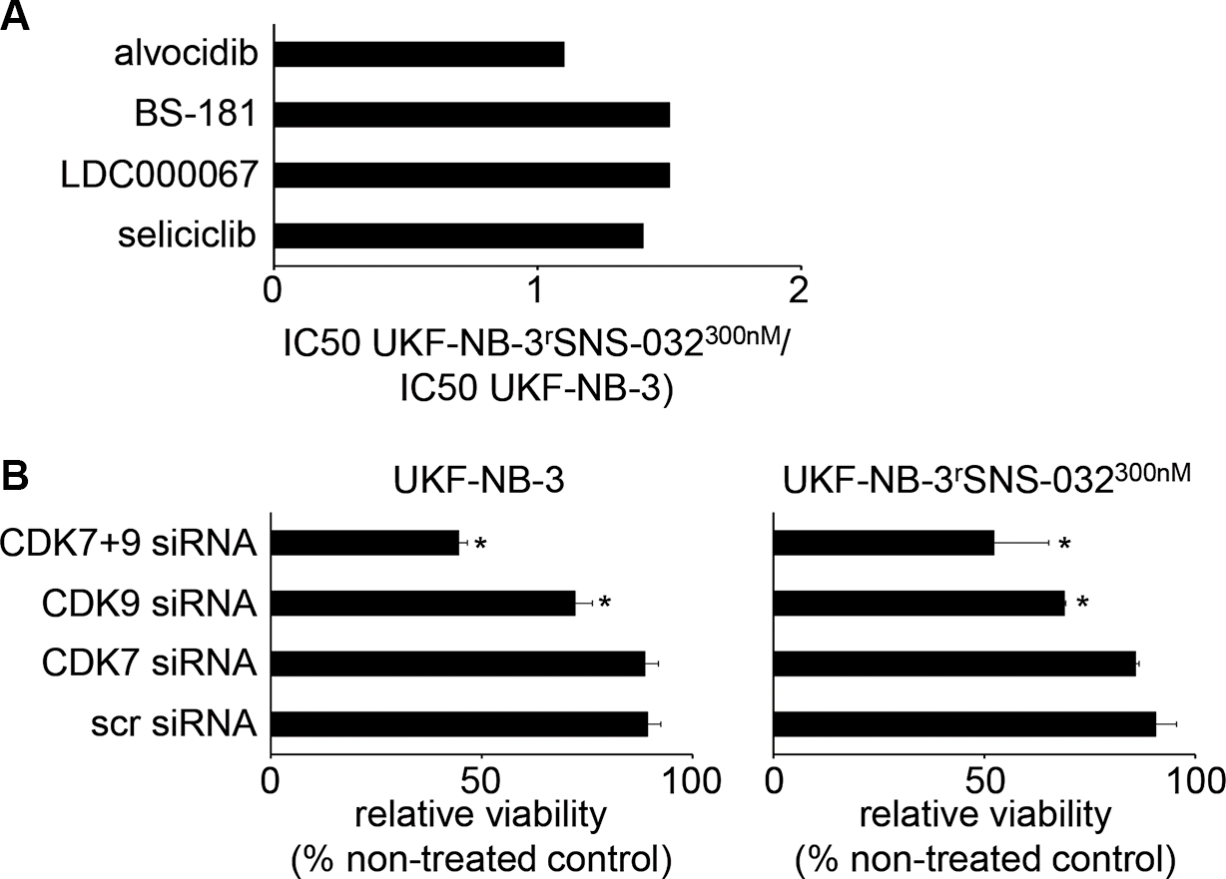
Sensitivity of UKF-NB-3 and its sub-line with acquired resistance to SNS-032 (UKF-NB-3^r^SNS-032^300nM^) to CDK inhibition by alternative inhibitors or by siRNA-mediated CDK depletion (**A**) Relative sensitivity of UKF-NB-3 and UKF-NB-3^r^SNS-032^300nM^ to the CDK2, 7, and 9 inhibitor seliciclib, the CDK7 inhibitor LDC000067, the CDK9 inhibitor BS-181, or the CDK 1,2,4,6,7, and 9 inhibitor alvocidib. Numerical values are presented in Supplementary Table S1F. (**B**) Effects of siRNA-mediated depletion of CDK7, CDK9, or CDK7 and CDK9 on UKF-NB-3 and UKF-NB-3^r^SNS-032^300nM^ cell viability as determined by MTT assay 72 h post-transfection. Non-targeting ‘scrambled’ siRNA (scr siRNA) served as control. Western blots indicating siRNA-mediated effects on protein levels are presented in Supplementary Figure S6. Numerical values are presented in Supplementary Table S1G. * *P* < 0.05 relative to scr siRNA.

Moreover, the relative resistances IC_50_ UKF-NB-3^r^SNS-032^300nM^/ IC_50_ UKF-NB-3 were ≤ 1.5 for the CDK2, 7, and 9 inhibitor seliciclib (also known as roscovitine or CYC202) [24], the CDK9 inhibitor LDC000067 [25], the CDK7 inhibitor BS-181 [26], and the CDK 1, 2, 4, 6, 7, and 9 inhibitor alvocidib (also known as flavopiridol or HMR-1275) [27] (Figure 6A; Supplementary Table S1F). Finally, UKF-NB-3^r^SNS-032^300nM^ cells were similar sensitive to siRNA-mediated depletion of CDK7 and CDK9 as UKF-NB-3 cells (Figure 6B, Supplementary Table S1G). Hence, UKF-NB-3^r^SNS-032^300nM^ cells do not seem to have acquired specific resistance to CDK inhibition.

### Investigation of SHEPrSNS-032^2000nM^, a SNS-032-resistant sub-line of the neuroblastoma cell line SHEP

In order to investigate to which extent ABCB1 expression may be a resistance mechanism in an additional model of acquired SNS-032 resistance, we established an SNS-032-resistant SHEP sub-line (SHEP^r^SNS-032^2000nM^). In contrast to the MYCN-amplified neuroblastoma cell line UKF-NB-3, SHEP cells do not harbor a MYCN amplification. Also in contrast to UKF-NB-3, SHEP cells express ABCB1 and display a higher SNS-032 IC_50_ value than UKF-NB-3 cells (SHEP 912 nM; UKF-NB-3 153 nM (Supplementary Table S1A, S1H)). SHEP cells were adapted to growth in the presence of SNS-032 2000 nM by step-wise increase of the SNS-032 concentration. No pre-existing SNS-032-resistant sub-population could be selected by directly applying SNS-032 2000 nM.

SHEP and SHEP^r^SNS-032^2000nM^ cells displayed similar doubling times (SHEP: 17.9 ± 1.4 h, SHEP^r^SNS-032^2000nM^ 17.9 ± 0.6 h) and a similar morphology (Supplementary Figure S1). Adaptation of SHEP cells to SNS-032 resulted in a further increase of the cellular ABCB1 levels but did not affect ABCG2 or ABCC1 expression levels (Figure 1, Supplementary Figure S2).

**Figure 7:**
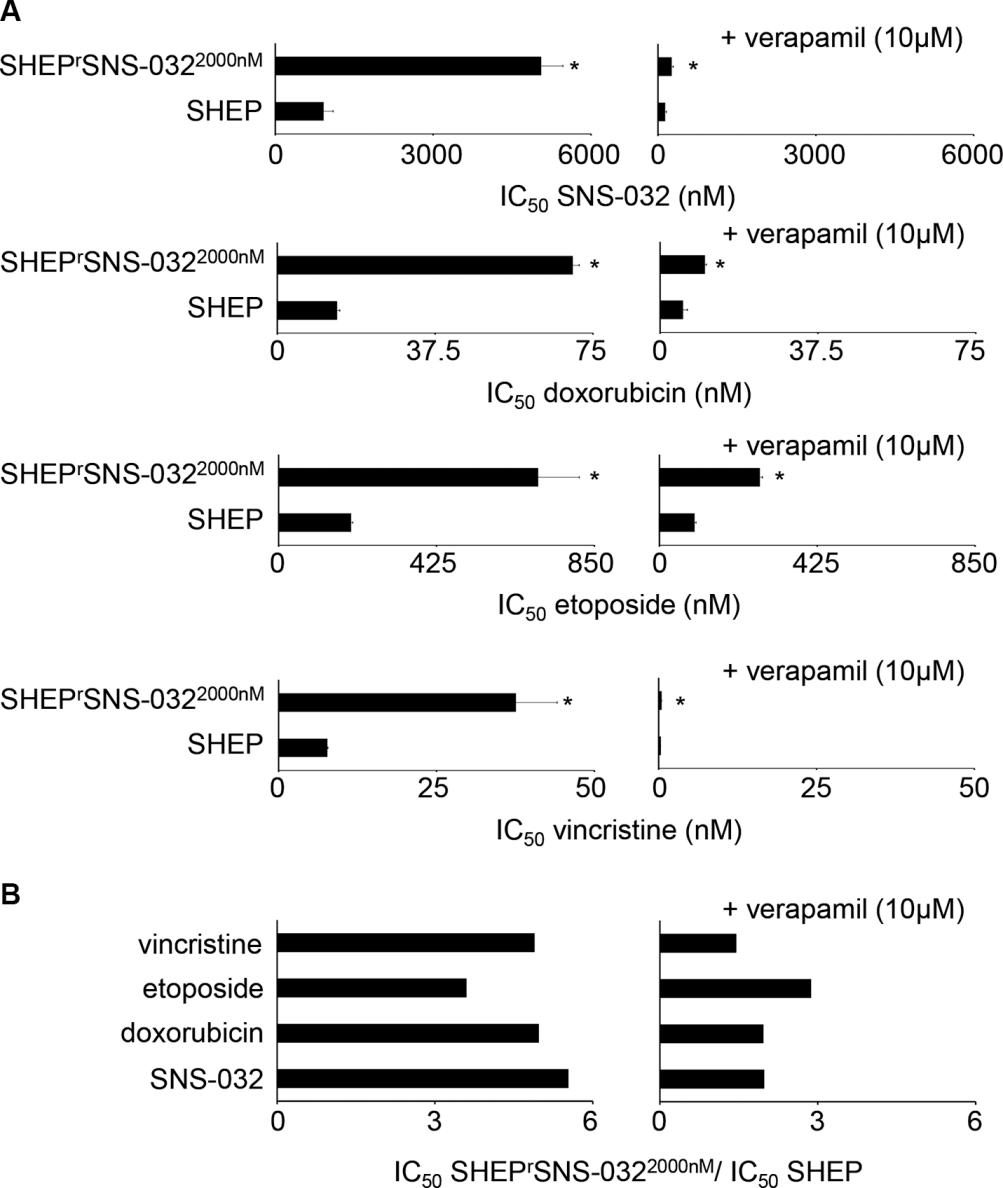
Sensitivity of SHEP and its sub-line with acquired resistance to SNS-032 (SHEP^r^SNS-032^2000nM^) to the cytotoxic ABCB1 substrates SNS-032, doxorubicin, etoposide, and vincristine (**A**) Concentrations that reduce cell viability by 50% (IC_50_) in the absence or presence of the ABCB1 inhibitor verapamil. Verapamil alone did not influence cell viability. * *P* < 0.05 relative to the drug concentration that reduces cell viability by 50% (IC_50_) in SHEP cells; (**B**) Fold change IC_50_ SHEP^r^SNS-032^2000nM^/ IC_50_ SHEP in the absence or presence of verapamil. Numerical values are presented in Supplementary Table S1H.

**Figure 8:**
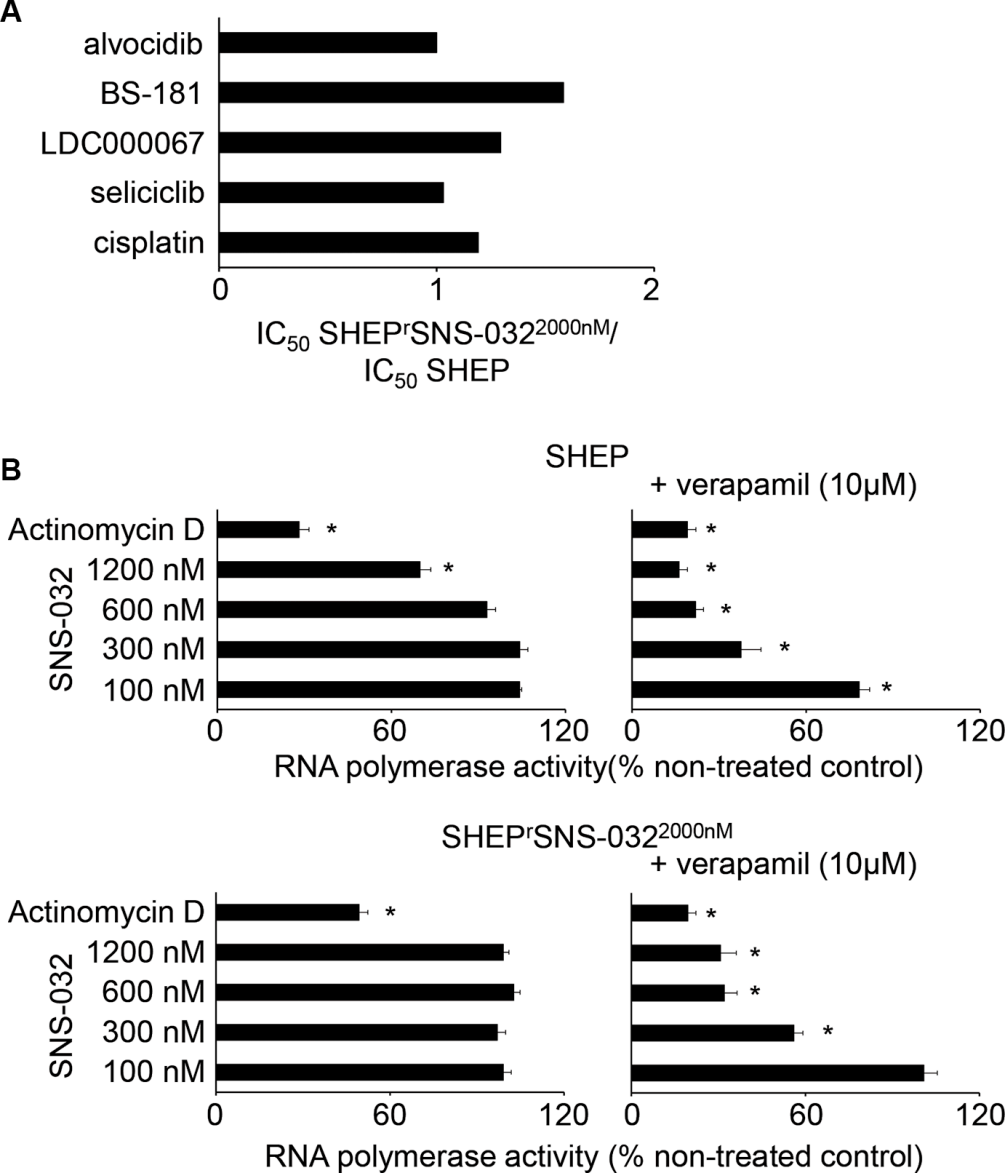
Sensitivity of SHEP and its sub-line with acquired resistance to SNS-032 (SHEP^r^SNS-032^2000nM^) to the non-ABCB1 substrates cisplatin, seliciclib (CDK2, 7, and 9 inhibitor), LDC000067 (CDK7 inhibitor), and BS-181 (CDK9 inhibitor) (**A**) Fold change IC_50_ SHEP^r^SNS-032^2000nM^/ IC_50_ SHEP; numerical values are presented in Supplementary Table S1K. (**B**) Effects of SNS-032 or actinomycin D (100 ng/mL) (interferes with RNA polymerase activity through DNA intercalation independently of CDK7 and CDK9) on RNA polymerase activity in SHEP and SHEP^r^SNS-032^2000nM^ cells in the absence or presence of the ABCB1 inhibitor verapamil (10 μM) as determined after 6 h of incubation. Numerical values are presented in Supplementary Table S1J. * *P* < 0.05 relative to untreated control

Similar to UKF-NB-3^r^SNS-032^300nM^ cells, (cross-)resistances predominantly depended on ABCB1 function in SHEP^r^SNS-032^2000nM^ cells (Figure 7 and Figure 8A, Supplementary Table S1H and Supplementary Table S1I). ABCB1 inhibition using verapamil did not always reduce the IC_50_ values for the ABCB1 substrates to the level of parental SHEP cells in the presence of verapamil. The fold change IC_50_ SHEP^r^SNS-032^2000nM^/ IC_50_ SHEP in the presence of verapamil was close to 2 for SNS-032 (1.98) and doxorubicin (1.96) and above 2 for etoposide (2.87), while it was 1.45 for vincristine (Figure 7, Supplementary Table S1H). For the non-ABCB1 substrate cisplatin and the CDK inhibitors seliciclib, LDC000067, BS-181, and alvocidib the fold changes IC_50_ SHEP^r^SNS-032^2000nM^/ IC_50_ SHEP were clearly below 2 (Figure 8A, Supplementary Table S1I).

In the presence of verapamil, SNS-032 600 nM caused maximum RNA polymerase inhibition in SHEP^r^SNS-032^2000nM^ cells (32 ± 4% activity relative to non-treated control). This effect was not further enhanced by increasing the SNS-032 concentration to 1200 nM (31 ± 5% activity relative to non-treated control) (Figure 8B, Supplementary Table S1J). In contrast to this, combined SHEP cell treatment with SNS-032 600 nM and verapamil reduced RNA polymerase activity to 22 ± 3% relative to untreated control, which was further decreased by combined SNS-032 1200 nM and verapamil treatment to 16 ± 3% (Figure 8B, Supplementary Table S1J). The positive control actinomycin D 100 ng/mL, which is also an ABCB1 substrate [28] and interferes with RNA polymerase activity through DNA intercalation independently of CDK7 and CDK9 [29, 30], exerted the same activity in SHEP cells (19.1 ± 2.9% relative to untreated control) and SHEP^r^SNS-032^2000nM^ cells (19.5 ± 2.8% relative to untreated control) in the presence of verapamil (Figure 8B, Supplementary Table S1J). These findings suggest that SHEP^r^SNS-032^2000nM^ cells have developed mechanisms to specifically compensate SNS-032-mediated CDK7- and CDK9 inhibition.

## DISCUSSION

To study acquired SNS-032 resistance mechanisms, we established two SNS-032-resistant neuroblastoma cell lines. Elevated ABCB1 expression represented, depending on the investigated cell line model, a dominant or even exclusive acquired SNS-032 resistance mechanism. In the clinics, few neuroblastomas appear to express ABCB1 at diagnosis [31]. However, ABCB1 expression might represent an acquired resistance mechanism in neuroblastoma [32]. In addition, ABCB1 expression may be an SNS-032-associated acquired resistance mechanism in neuroblastoma. Drug-adapted cancer cell lines were previously successfully used to identify novel clinically relevant acquired drug resistance mechanisms [33–37].

The resistance status of the SNS-032-resistant UKF-NB-3 sub-line UKF-NB-3^r^SNS-032^300nM^ depended exclusively on ABCB1 function. ABCB1 inhibition completely re-sensitized UKF-NB-3^r^SNS-032^300nM^ to SNS-032 to the level of UKF-NB-3 cells. The same observation was made for UKF-NB-3^r^SNS-032^300nM^ cell sensitivity to the ABCB1 substrates doxorubicin, etoposide, and vincristine. UKF-NB-3^r^SNS-032^300nM^ cells displayed cross-resistance to these agents but their sensitivity to these drugs returned to the levels of UKF-NB-3 in the presence of the ABCB1 inhibitor verapamil. Moreover, UKF-NB-3^r^SNS-032^300nM^ cells did not display cross-resistance to the non-ABCB1 substrate cisplatin or the alternative CDK inhibitors seliciclib, LDC000067, BS-181, or alvocidib. Previous results had demonstrated that SNS-032 reduces the viability of UKF-NB-3 cells (and other cancer cells) via interference with CDK7 and CDK9 and subsequent RNA polymerase II inhibition resulting in depletion of anti-apoptotic proteins with a high turnover rate including Mcl-1, XIAP, and survivin [7, 8, 19]. In the presence of the ABCB1 inhibitor verapamil, SNS-032 exerted similar effects on CDK7, CDK9, and RNA polymerase II activity as well as on the cellular levels of Mcl-1, XIAP, and survivin in UKF-NB-3^r^SNS-032^300nM^ and in UKF-NB-3 cells. Moreover, UKF-NB-3^r^SNS-032^300nM^ and UKF-NB-3 cells did not differ in their sensitivity to siRNA-mediated depletion of CDK7 and CDK9. Taken together, these findings demonstrate that ABCB1 expression represents an exclusive resistance mechanism in UKF-NB-3^r^SNS-032^300nM^ cells.

ABCB1 expression as sole resistance mechanism is unusual even among UKF-NB-3 sub-lines with acquired resistance to ABCB1 substrates. The ABCB1-expressing drug-resistant UKF-NB-3 sub-lines UKF-NB-3^r^DOX^20^, UKF-NB-3^r^ETO^100^, and UKF-NB-3^r^VCR^10^ maintained a substantial level of resistance to the respective drug of adaptation even when ABCB1 was inhibited. In addition, UKF-NB-3^r^DOX^20^ and UKF-NB-3^r^VCR^10^ cells were (in contrast to UKF-NB-3^r^SNS-032^300nM^ cells) less sensitive to the non-ABCB1 substrate cisplatin than UKF-NB-3 cells. These findings demonstrate that UKF-NB-3^r^DOX^20^, UKF-NB-3^r^VCR^10^, and UKF-NB-3^r^ETO^100^ cells have developed further resistance mechanisms in addition to increased ABCB1 expression.

The investigation of the second SNS-032-resistant neuroblastoma cell line SHEP^r^SNS-032^2000nM^ resulted in slight but noticeable differences. We had selected the neuroblastoma cell line SHEP as additional model for our study because it differs in two crucial parameters from UKF-NB-3: 1) SHEP cells do not harbor a MYCN amplification, a major marker of high-risk neuroblastoma [1,2], and 2) SHEP cells are characterized by intrinsic ABCB1 expression. SHEP^r^SNS-032^2000nM^ cells retained some low-level resistance to SNS-032 and doxorubicin (about 2-fold decreased sensitivity compared to SHEP) and some more pronounced resistance to etoposide (2.9-fold relative to SHEP) also in the presence of verapamil. This demonstrates that SHEP^r^SNS-032^2000nM^ cells have acquired other resistance mechanisms in addition to ABCB1 expression. SNS-032 also exerted decreased effects on RNA polymerase activity in SHEP^r^SNS-032^2000nM^ cells than in SHEP cells when ABCB1 was inhibited. However, both cell lines displayed in the presence of the ABCB1 inhibitor verapamil the same sensitivity to RNA polymerase inhibition by actinomycin D that is also an ABCB1 substrate [28] and interferes with RNA polymerase activity by CDK7- and CDK9-independent mechanisms [29, 30]. These findings are again in contrast to observations in UKF-NB-3^r^SNS-032^300nM^ cells and indicate that SHEP^r^SNS-032^2000nM^ cells developed mechanisms to bypass SNS-032-induced CDK7 and 9 inhibition and subsequent RNA polymerase activity but no general resistance to RNA polymerase inhibition. This specific resistance to RNA polymerase inhibition via interference with CDK7 and 9 also confirms CDK7 and 9 as critical drug targets of SNS-032 in neuroblastoma that was previously suggested [19]. Hence, drug-adapted cells can be used to identify and confirm drug mechanisms of action.

A likely explanation for the differences observed between the resistance phenotypes of UKF-NB-3^r^SNS-032^300nM^ and SHEP^r^SNS-032^2000nM^ cells is that SHEP^r^SNS-032^2000nM^ cells were adapted to higher SNS-032 concentrations. While non-ABCB1 expressing UKF-NB-3 cells are highly sensitive to SNS-032 (IC_50_ 153 nM), ABCB1-expressing SHEP cells display an IC_50_ value of 912 nM, which is above the therapeutically achievable SNS-032 plasma concentration of 754 nM [14]. Therefore, SHEP cells were adapted to growth in the presence of a much higher SNS-032 concentration (2000 nM) than UKF-NB-3 cells (300 nM). The SNS-032 IC_50_ of UKF-NB-3^r^SNS-032^300nM^ cells (607 nM) remained below the SNS-032 IC_50_ of parental SHEP cells, whereas the SNS-032 IC_50_ of SHEP^r^SNS-032^2000nM^ cells (5045 nM) was about 8-fold higher than that of UKF-NB-3^r^SNS-032^300nM^ cells. Thus, it does not appear implausible that UKF-NB-3^r^SNS-032^300nM^ cells might develop additional resistance mechanisms if they were further adapted to higher SNS-032 concentrations.

It remains unclear why SHEP^r^SNS-032^2000nM^ cells did not display cross-resistance to the other CDK inhibitors investigated. It is known that kinase inhibitors designed to interfere with the same or similar targets may substantially differ in their overall pharmacological profiles (with regards to kinase inhibition as well as other structures) [8, 10, 38–42]. Hence, the lack of cross-resistance may not be too surprising. The inhibitory profiles of the different CDK inhibitors differ with regard to their CDK inhibitory profiles. Seliciclib interferes (in contrast to SNS-032) with CDK5 in addition to CDK2, CDK7, and CDK9 [24, 43]. BS-181 does in contrast to SNS-032 not inhibit CDK9 [26]. LDC000067 was introduced as selective CDK9 inhibitor with negligible affinity to alternative CDKs [25]. Alvocidib is a broad spectrum CDK inhibitor acting on CDKs 1, 2, 4, 6, 7, and 9 [27].

Notably, cisplatin-resistant (UKF-NB-3^r^CDDP^1000^), doxorubicin-resistant (UKF-NB-3^r^DOX^20^), and vincristine-resistant (UKF-NB-3^r^VCR^10^) UKF-NB-3 sub-lines remained sensitive to seliciclib, LDC000067, BS-181, and alvocidib (Supplementary Table S1K) further supporting a possible role of CDKs as drug targets in neuroblastoma including therapy-refractory disease as previously suggested [17–19]. The alvocidib IC_50_s were below 400 nM. In phase I clinical trials, steady-state plasma alvocidib concentrations > 400 μM were achieved in human patients [44].

In conclusion, we show that ABCB1 expression represents the predominant resistance mechanism in neuroblastoma cells with acquired resistance to SNS-032. Most strikingly (and in clear contrast to neuroblastoma cell lines with acquired resistance to other anti-cancer agents that are ABCB1 substrates), ABCB1 expression is an exclusive resistance mechanism in UKF-NB-3^r^SNS-032^300nM^ cells and a predominant resistance mechanism in SHEP^r^SNS-032^2000nM^ cells. Although selective ABCB1 inhibitors are available [45, 46], clinical trials were disappointing. Reasons included that ABCB1 transporter inhibition may affect ABCB1-expressing hematopoietic stem cells and the body distribution of drugs due to effects on ABCB1 present on tissue barriers (e.g. the blood-brain-barrier). Also cancer cells may express multiple ABC transporters [45, 46]. However, ABCB1 expression represents the dominant (sometimes exclusive) resistance mechanism to SNS-032 in neuroblastoma. Therefore, ABCB1 inhibitors may be candidates for combination therapy with SNS-032 that increase SNS-032 efficacy through (re)sensitization of ABCB1-expressing cancer cells, possibly reducing resistance formation.

## MATERIALS AND METHODS

### Drugs

SNS-032, BS-181, and LDC000067 were purchased from Selleck Chemicals via BIOZOL GmbH (Eching, Germany), seliciclib from LC Laboratories (Woburn, Massachusetts), vincristine, cisplatin, and etoposide from TEVA GmbH (Radebeul, Germany), actinomycin D from Lundbeck Pharmaceuticals Ireland Limited (Dublin, Ireland), and verapamil from Sigma-Aldrich (Munich, Germany).

### Synthesis and characterisation of SNS-032-BODIPY

SNS-032-BODIPY was synthesized using SNS-032 (Selleck Chemicals) and BDP-FL-NHS-ester (Lumiprobe, Hannover, Germany) (Supplementary Figure S7A). All other reagents and solvents were purchased from Fisher Scientific (Loughborough, UK). NMR spectra were collected on a JEOL 400 MHz. HRMS were collected on a Bruker MicroTOFQ spectrometer by direct injection with a MeOH/HCOOH matrix. Excitation and emission spectra were collected on an Edinburgh Instruments FS-5 spectrofluorimeter. HPLC traces were collected on a Dionex U3000 HPLC apparatus.

To 7.68 mg (2.02 × 10^-2^ mmol) of **SNS-032** chloroform (1.3 mL) was added and the mixture sonicated until dossolution. **BDP-FL NHS ester** (purchased from Lumiprobe; 8.09 mg, 2.08 × 10^-2^ mmol) was also dissolved in chloroform (1.3mL) and sonicated to ensure dissolution. The SNS-032 mixture was added to BDP-FL and the resulting sample was sonicated for 1 h. Reaction was monitored using thin-layer chromatography (dichloromethane: methanol – 4:0.1; product *rf*: 0.21). Reaction mixture was concentrated and then purified by preparative TLC using the above noted eluent. The adsorbed product band on the silica plate was scraped, isolated and rinsed repeatedly with acetonitrile to extract the product yielding 9.0 mg (1.38 × 10^-2^ mmol; 68%). Purity was confirmed by reverse-phase HPLC (Figure 7B–7E).

^1^H NMR (400 MHz, CDCl_3_): d 7.33 (s, 1H, Ar-H), 7.13 (s, 1H, Ar-H), 6.93 (d, *J* = 4.2 Hz, 1H, Ar-H), 6.61 (s, 1H, Ar-H), 6.35 (d, *J* = 4.2 Hz, 1H, Ar-H), 6.11 (s, 1H, Csp2-H), 5.33 (bs, 1H, NH), 3.96 (s, 2H), 4.05 (bd, *J* = 15.1 Hz, 2H), 3.32 (t, *J* = 7.8 Hz, 2H), 3.12 (t, *J* = 15.1 Hz, 2H), 2.80 (t, *J* = 7.8 Hz, 2H), 2.63 (m, 1H), 2.58 (s, 3H), 2.27 (s, 3H), 1.96 (m, 2H), 1.73 (m, 2H). ^19^F NMR (376 MHz, DMSO-*d_6_*): −145.675 (q, *J*_F-B_ = 31.4 Hz). ^11^B NMR (128 MHz, DMSO-*d_6_*): −1.7 (t, *J*_B-F_ = 31 Hz) HRMS: *m/z* (M + H) calcd. = 655.2508, found = 655.2562; *m/z* (M-^19^F) calcd. = 635.2446, found = 635.2492.

### Cell lines

The MYCN-amplified neuroblastoma cell line UKF-NB-3 was established from a stage 4 neuroblastoma patient [44]. SHEP cells [45] were kindly provided by Dr. Angelika Eggert (Universität Duisburg-Essen, Germany). Neuroblastoma cell lines were adapted to growth in the presence of anti-cancer drugs by continuous exposure to increasing drug concentrations as described previously [19, 47, 49]. All neuroblastoma cell lines with acquired drug resistance were derived from the resistant cancer cell line (RCCL) collection. The corresponding IC50 values for the parental cells and their drug-resistant sub-lines were provided previously [19, 50]. All cells were propagated in IMDM supplemented with 10 % fetal calf serum (FCS), 100 IU/ml penicillin, and 100 μg/ml streptomycin at 37°C. Cells were routinely tested for mycoplasma contamination and authenticated by short tandem repeat profiling.

UKF-NB-3 cells were transduced with lentiviral vectors encoding for ABCB1 (also known as MDR1 or P-glycoprotein) or ABCG2 (also known as BCRP) as described previously [50, 51] using the Lentiviral Gene Ontology (LeGO) vector technology [52] (www.lentigo-vectors.de).

### Viability assay

Cell viability was tested either by the 3-(4,5-dimethylthiazol-2-yl)-2,5-diphenyltetrazolium bromide (MTT) dye reduction assay after 120 h incubation modified as described previously [47, 50].

### Determination of ABCB1, ABCG2, and ABCC1 expression

The ABC-transporters ABCB1, ABCC1, and ABCG2 were detected by flow cytometry as described previously [51] using specific primary antibodies against ABCB1 (Alexis Biochemicals via AXXORA Deutschland, Lörrach, Germany), ABCC1, and ABCG2 (Kamiya Biomedical Company, Seattle, Washington) and secondary phycoerythrin(PE)-labelled goat anti-mouse antibody (PE, R&D Systems, Wiesbaden, Germany).

### RNA interference experiments

Synthetic siRNAoligonucletides targeting CDK7, CDK9, ABCC1, or ABCB1 (ON-TARGETplusSMARTpoolsiRNAs) were purchased from Dharmacon (Lafayette, CO, USA). The non-targeting siRNA ON-TARGETplusSMARTpool (Dharmacon) was used as negative control. Transfections were performed using the Neon™ Transfection System (Invitrogen, Darmstadt, Germany) according to the manufacturer's protocol. UKF-NB-3 cells or UKF-NB-3^r^SNS-032^300nM^ cells were grown to about 60-80% confluence, trypsinized and 2 × 10^6^ cells were re-suspended in 200 μl of resuspension buffer containing 2.5 μM siRNA. Electroporation was performed in a pipette tip chamber with previously optimized adjustments (voltage 1400, width 20, 2 pulses). After electroporation, the cells were transferred into fibronectin (100 μg/ml)–coated well plates containing pre-warmed IMDM plus 10% FCS.

### Western blot

Cells were lysed in Triton X-sample buffer and separated by SDS-PAGE. Proteins were detected using specific antibodies directed against β-actin (BioVision via BioCat GmbH, Heidelberg, Germany), ABCC1, ABCG2 (both from Santa Cruz Biotechnology, Heidelberg, Germany), ABCB1, XIAP, Mcl-1, CDK7, CDK9 (all from Cell Signaling via New England Biolabs, Frankfurt am Main, Germany), RNA polymerase II, Ser2-phosphorylated RNA polymerase II, Ser5-phosphorylated RNA polymerase II (all from Abcam, Cambridge, UK), and survivin (R&D Systems, Wiesbaden, Germany). Protein bands were visualized by enhanced chemiluminescence using a commercially available kit (Thermo Scientific, Schwerte, Germany).

### Flow cytometry

Cells were incubated with SNS-032-BODIPY for 45 min at 37°C. Then, the cells were washed twice with PBS before fresh medium was added. The cellular fluorescence was analysed after a further 120 minutes using a FACS Canto (BD Biosciences, Heidelberg, Germany) using the FL1 channel. In verapamil-treated cells, verapamil was continuously present throughout the experiments including a pre-incubation period of 30 minutes at 37°C prior to the addition of SNS-032-BODIPY.

### RNA synthesis assay

Detection of global RNA synthesis was performed as previously described [19] using the Click-iT^®^ RNA HCS Assay (Invitrogen, Darmstadt, Germany).

## SUPPLEMENTARY MATERIALS FIGURES AND TABLES




