# Supplementary material for: ABCB1 as predominant resistance mechanism in cells with acquired SNS-032 resistance
Source: Oncotarget. 2016 Aug 9;7(36):58051–64. doi: 10.18632/oncotarget.11160 (PMC5295411; doi:10.18632/oncotarget.11160)
Supplement: Supplementary file 1 [file oncotarget-07-58051-s001.pdf]

ABCB1 as predominant resistance mechanism in cells with acquired SNS-032 resistance

Supplementary Materials

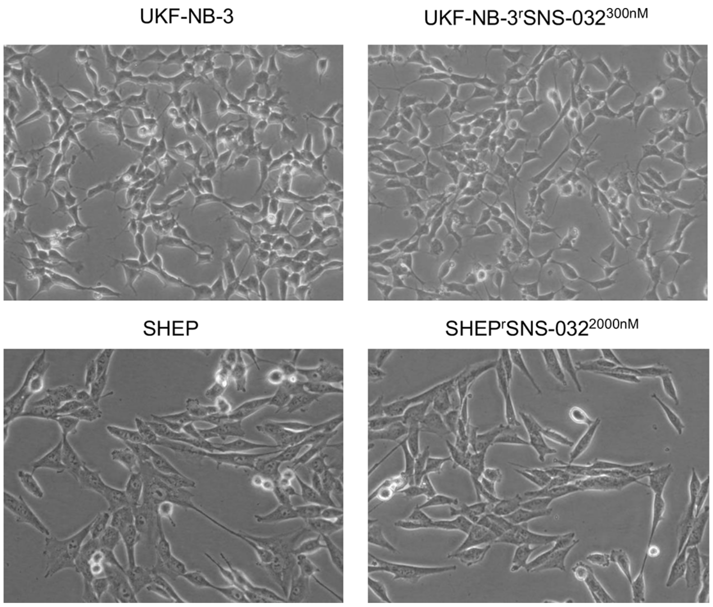

Supplementary Figure S1: Representative photographs showing the morphology of UKF-NB-3, UKF-NB-3rSNS-032^{300nM}, SHEP, and SHEP^{r}SNS-032^{2000nM} cells.

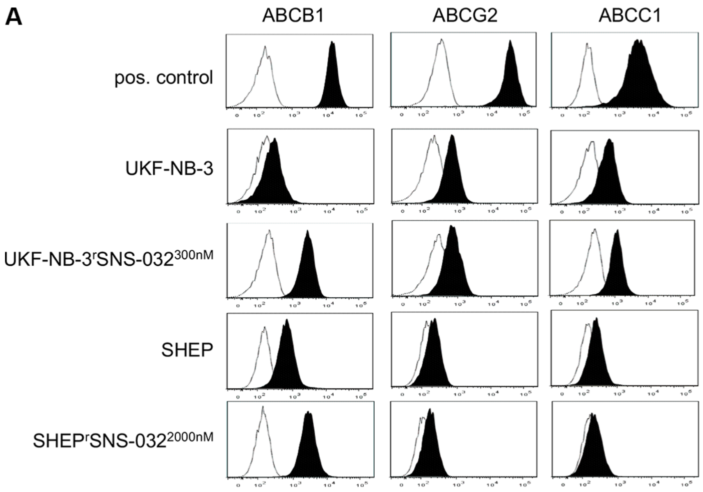

Supplementary Figure S2: Representative flow cytometry histograms indicating ABCB1, ABCG2, and ABCC1 protein levels in UKF-NB-3, UKF-NB-3^{r}SNS-032^{300nM}, SHEP, and SHEP^{r}SNS-032^{2000nM} cells. Positive controls were ABCB1- transduced UKF-NB-3 cells for ABCB1, ABCG2-transduced UKF-NB-3 cells for ABCG2, and NLF^{r}VCR^{10} cells for ABCC1. White peaks indicate isotype controls, black peaks indicate staining by ABC transporter-specific antibodies.

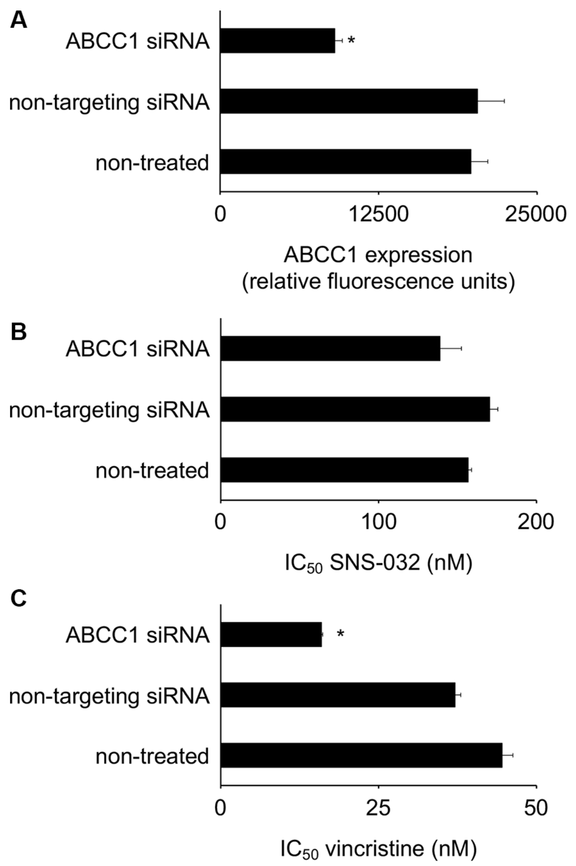

Supplementary Figure S3: SNS-032 activity is not affected by ABCC1 expression. (**A**) siRNA-mediated depletion of ABCC1 in NLF^{r}VCR^{10} cells (ABCC1 expression in NLF^{r}VCR^{10} cells is shown in Figure 1 and Supplementary Figure S2). (**B**) siRNA-mediated ABCC1 depletion does not affect the SNS-032 concentration that reduces NLF^{r}VCR^{10} cell viability by 50% (IC_{50}) as indicated by MTT after 5 days of incubation. (**C**) siRNA-mediated ABCC1 depletion reduces the vincristine IC_{50} in NLF^{r}VCR^{10} cells. * $P < 0.05$ relative to non-treated control.

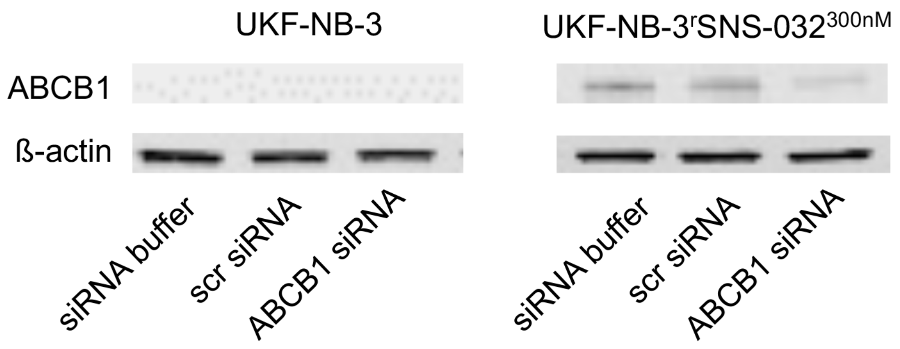

Supplementary Figure S4: Effects of siRNA directed against ABCB1 on the ABCB1 protein levels as indicated by Western blot. Non-targeting “scrambled” siRNA (scr siRNA) served as control. Blots are cropped.

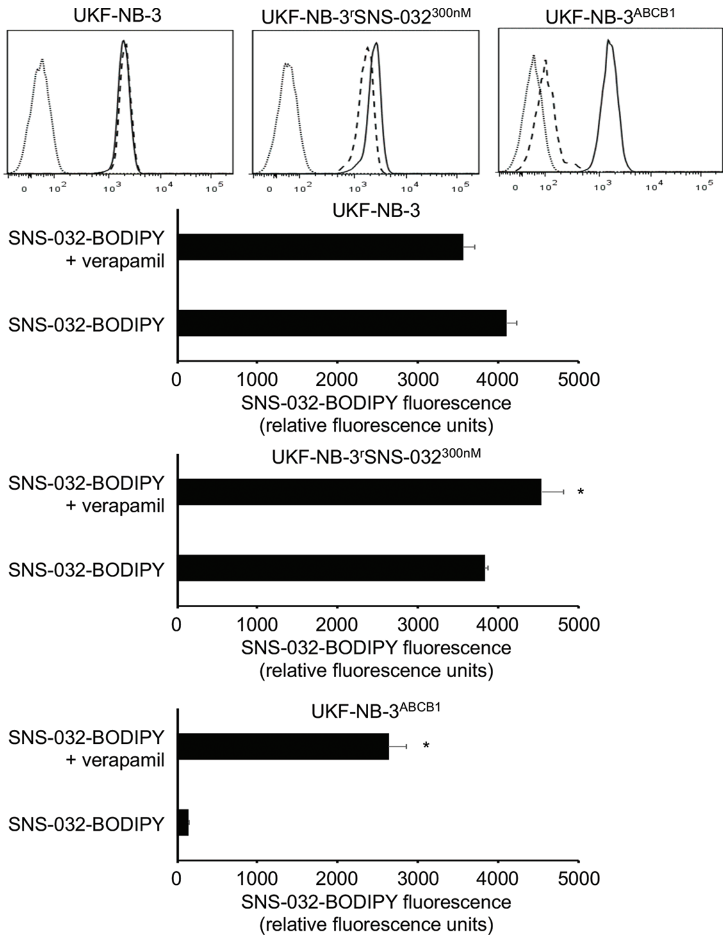

Supplementary Figure S5: Representative histograms (dotted line, untreated; dashed line SNS-032- BODIPY; solid line, SNS-032-BODIPY plus verapamil) and quantification of SNS-032-BODIPY (100 nM) fluorescence in neuroblastoma cells in the absence or presence of verapamil (10 µM) as indicated by flow cytometry. *$P < 0.05$ compared to SNS-032-BODIPY.

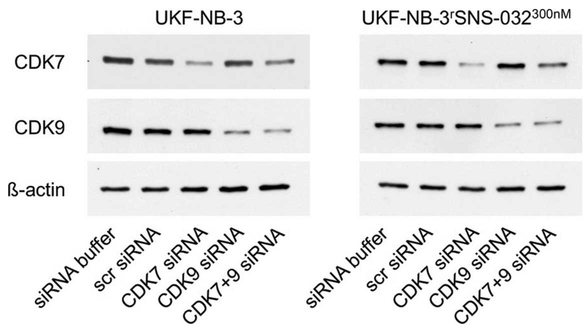

Supplementary Figure S6: Effects of siRNA directed against CDK7 and/or CDK9 on the respective protein levels as indicated by Western blot. Non-targeting "scrambled" siRNA (scr siRNA) served as control. Blots are cropped.

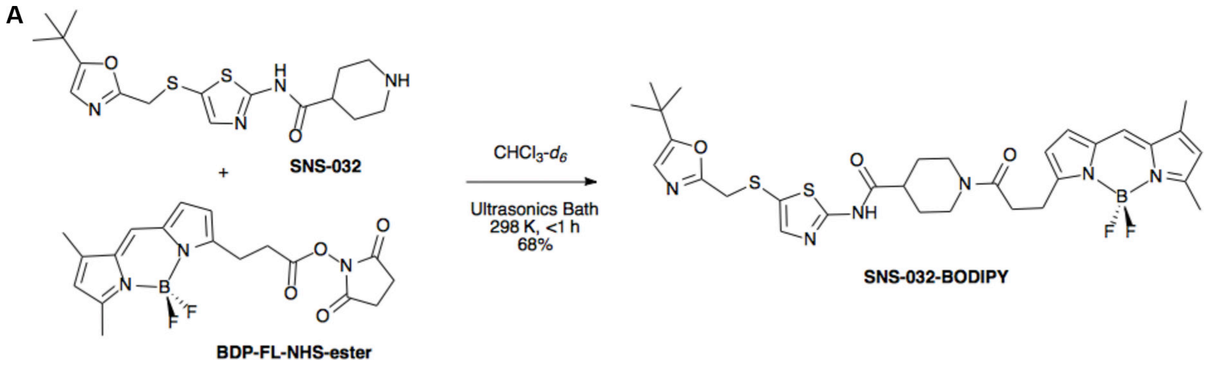

Supplementary Figure S7A: Overview of the synthesis of SNS-032-BODIPY.

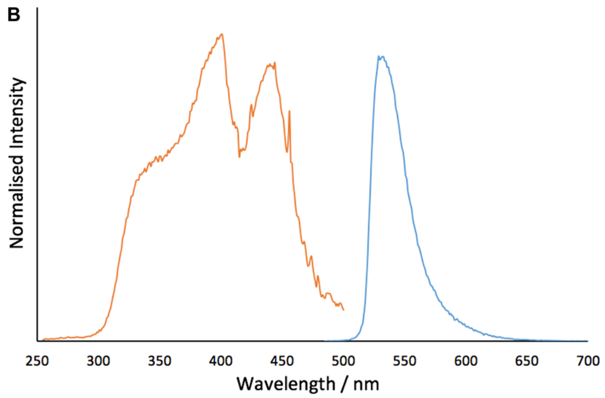

Supplementary Figure S7B: Normalized excitation spectrum, Orange (λ_{em} = 529 nm) and emission Spectrum, Blue (λ_{ex} = 396 nm) of SNS-032-BODIPY in THF at 298 K.

C

Column	Phenomenex Luna C8 5u 2.0 x 30 mm
Temperature of column	40°C
Mobile Phase	A = Water + 0.2% H_3PO_4<br>B = MeOH + 0.2% H_3PO_4
Gradient	10-100% B over 2.5mins @ 3ml/min
DAD	DAD 220, **254**, 270, 310 nm
Machine	Dionex U3000 HPLC

Supplementary Figure S7C: HPLC conditions for the purification of SNS-032-BODIPY.

D

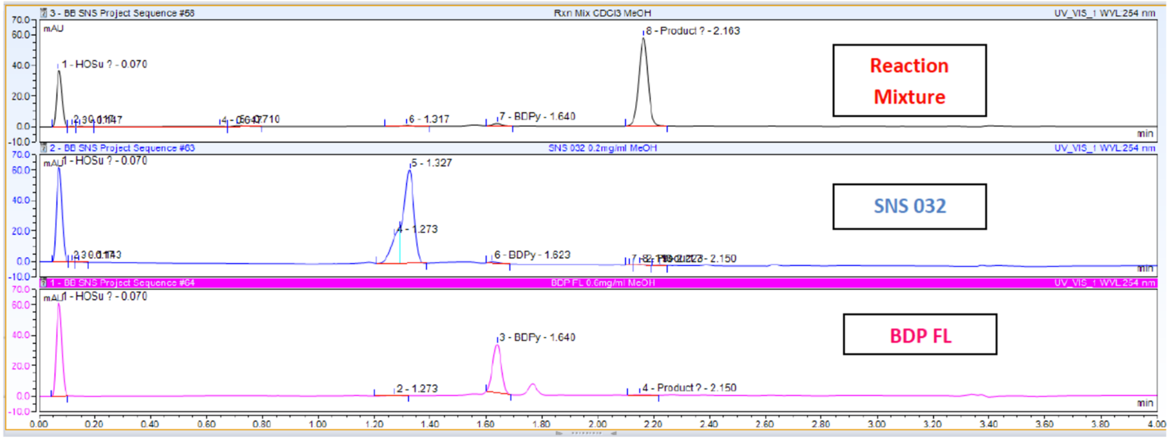

Supplementary Figure S7D: HPLC traces of reaction mixture for synthesis of SNS-032-BODIPY and both starting materials; note product peak at tR = 2.163 min.

E

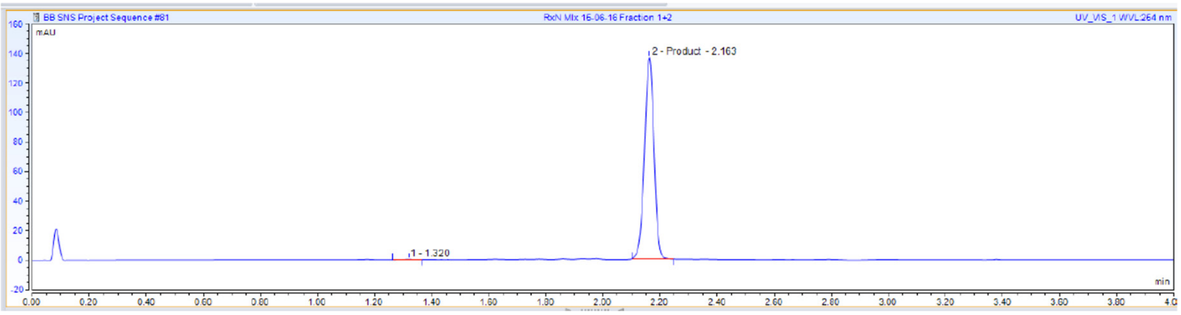

Supplementary Figure S7E: HPLC trace of purified SNS-032-BODIPY demonstrating pure sample at *t*R = 2.163 min.

Supplementary Table S1A. Effects of the ABCB1 substrates SNS-032, doxorubicin, etoposide, and vincristine on the viability of the neuroblastoma cell line UKF-NB-3 and its sub-lines with acquired resistance to SNS-032 (UKF-NB-3^{r}SNS-032^{300nM}), doxorubicin (UKF-NB-3^{r}DOX^{20}), etoposide (UKF-NB-3^{r}ETO^{100}), or vincristine (UKF-NB-3^{r}VCR^{10}). See Supplementary_Table_S1A

Supplementary Table S1B. Effects of siRNA-mediated ABCB1 depletion on SNS-032 sensitivity in UKF-NB-3 and UKF-NB-3rSNS-032^{300nM} cells. See Supplementary_Table_S1B

Supplementary Table S1C. Effects of the ABCB1 inhibitor zosuquidar on the SNS-032 sensitivity of UKF-NB-3 and UKF-NB-3^{r}SNS-032^{300nM} cells. See Supplementary_Table_S1C

Supplementary Table S1D. Effects of the non-ABCB1 substrate cisplatin on the viability of the neuroblastoma cell line UKF-NB-3 and its sub-lines with acquired resistance to SNS-032 (UKF-NB-3^{r}SNS-032^{300nM}), cisplatin (UKF-NB-3^{r}CDDP^{1000}), doxorubicin (UKF-NB-3^{r}DOX^{20}), etoposide (UKF-NB-3^{r}ETO^{100}), or vincristine (UKF-NB-3rVCR10). See Supplementary_Table_S1D

Supplementary Table S1E. Effects of SNS-032, doxorubicin, etoposide, and vincristine on the viability of the neuroblastoma cell line UKF-NB-3 and its sub-line with acquired resistance to cisplatin (UKF-NB-3^{r}CDDP^{1000}). See Supplementary_Table_S1E

Supplementary Table S1F. Effects of the CDK2, 7, and 9 inhibitor seliciclib, the CDK9 inhibitor LDC000067, the CDK7 inhibitor BS-181, and the CDK 1,2,4,6,7, and 9 inhibitor alvocidib on the viability of the neuroblastoma cell line UKF-NB-3 and its sub-line with acquired resistance to SNS- 032 (UKF-NB-3^{r}SNS-032^{300nM}). See Supplementary_Table_S1F

Supplementary Table S1G. Effects of siRNA-mediated depletion of CDK7, CDK9, and CDK7 and CDK9 on the viability of the neuroblastoma cell line UKF-NB-3 and its sub-line with acquired resistance to SNS-032 (UKF-NB-3^{r}SNS-032^{300nM}) as determined by MTT assay 72 h post-transfection. See Supplementary_Table_S1G

Supplementary Table S1H. Effects of the ABCB1 substrates SNS-032, doxorubicin, etoposide, and vincristine on the viability of the neuroblastoma cell line SHEP and its sub-line with acquired resistance to SNS-032 (SHEP^{r}SNS-032^{2000nM}). See Supplementary_Table_S1H

Supplementary Table S1I. Effects of the non-ABCB1 substrate cisplatin, the CDK2, 7, and 9 inhibitor seliciclib, the CDK9 inhibitor LDC000067, the CDK7 inhibitor BS-181, and the CDK 1,2,4,6,7, and 9 inhibitor alvocidib on the viability of the neuroblastoma cell line SHEP and its sub-line with acquired resistance to SNS-032 (SHEP^{r}SNS-032^{2000nM}). See Supplementary_Table_S1I

Supplementary Table S1J. Effects of SNS-032 or actinomycin D on the RNA polymerase activity in the neuroblastoma cell line SHEP and its sub-line with acquired resistance to SNS-032 (SHEP^{r}SNS- 032^{2000nM}) in the absence or presence of the ABCB1 inhibitor verapamil (10 µM) after 6 h of incubation. See Supplementary_Table_S1J

Supplementary Table S1K. Effects of the the CDK2, 7, and 9 inhibitor seliciclib, the CDK9 inhibitor LDC000067, the CDK7 inhibitor BS-181, and the CDK 1,2,4,6,7, and 9 inhibitor alvocidib on the viability of UKF-NB-3 sub-lines with acquired resistance to cytotoxic anti-cancer drugs. See Supplementary_Table_S1K
