# Supplementary material for: ABCB1 as predominant resistance mechanism in cells with acquired SNS-032 resistance
Source: Oncotarget. 2016 Aug 9;7(36):58051–64. doi: 10.18632/oncotarget.11160 (PMC5295411; doi:10.18632/oncotarget.11160)
Supplement: Supplementary file 2 [file oncotarget-07-58051-s002.docx]

**Suppl. Table 1A**. Effects of the ABCB1 substrates SNS-032, doxorubicin, etoposide, and vincristine on the viability of the neuroblastoma cell line UKF-NB-3 and its sub-lines with acquired resistance to SNS-032 (UKF-NB-3^r^SNS-032^300nM^), doxorubicin (UKF-NB-3^r^DOX^20^), etoposide (UKF-NB-3^r^ETO^100^), or vincristine (UKF-NB-3^r^VCR^10^).

|  |  | + verapamil 10µM | |
| --- | --- | --- | --- |
| Cell line | IC_50_^1^ SNS-032 (nM) | verapamil alone^2^ (% control) | IC_50_ SNS-032 (nM) |
| UKF-NB-3 | 152.6 ± 32.8 | 88 ± 10 | 140.5 ± 41.6 |
| UKF-NB-3^r^SNS-032^300nM^ | 606.7 ± 16.4  (4.0)^3^ | 92 ± 15 | 162.4 ± 1.7  (1.1)^4^ |
| UKF-NB-3^r^DOX^20^ | 4284 ± 302  (28.1) | 98 ± 13 | 242.4 ± 39.4  (1.6) |
| UKF-NB-3^r^ETO^100^ | 338.1 ± 40.1  (2.2) | 94 ± 7 | 145.0 ± 29.4  (1.0) |
| UKF-NB-3^r^VCR^10^ | 725.7 ± 191.1  (4.8) | 97 ± 11 | 140.1 ± 10.5  (0.9) |
|  |  |  |  |
|  |  | + verapamil 10µM | |
| Cell line | IC_50_^1^ doxorubicin (nM) | verapamil alone^2^ (% control) | IC_50_ doxorubicin (nM) |
| UKF-NB-3 | 9.0 ± 2.6 | 88 ± 10 | 6.1 ± 1.5 |
| UKF-NB-3^r^SNS-032^300nM^ | 22 ± 6  (2.4)^3^ | 92 ± 15 | 6.1 ± 1.7  (0.7)^4^ |
| UKF-NB-3^r^DOX^20^ | 116 ± 13  (12.9) | 98 ± 13 | 20 ± 2  (2.2) |
| UKF-NB-3^r^ETO^100^ | 39 ± 4  (4.3) | 94 ± 7 | 24 ± 2  (2.7) |
| UKF-NB-3^r^VCR^10^ | 81 ± 6  (9.0) | 97 ± 11 | 18 ± 4  (2.0) |

|  |  | + verapamil 10µM | |
| --- | --- | --- | --- |
| Cell line | IC_50_^1^ etoposide (nM) | verapamil alone^2^ (% control) | IC_50_ etoposide (nM) |
| UKF-NB-3 | 70 ± 5 | 88 ± 10 | 66 ± 3 |
| UKF-NB-3^r^SNS-032^300nM^ | 143 ± 15  (2.0)^3^ | 92 ± 15 | 66 ± 22  (1.0)^4^ |
| UKF-NB-3^r^DOX^20^ | 1045 ± 58  (15.0) | 98 ± 13 | 359 ± 17  (5.1) |
| UKF-NB-3^r^ETO^100^ | 720 ± 41  (10.3) | 94 ± 7 | 449 ± 31  (6.4) |
| UKF-NB-3^r^VCR^10^ | 398 ± 37  (5.7) | 97 ± 11 | 216 ± 5  (3.1) |
|  |  |  |  |
|  |  | + verapamil 10µM | |
| Cell line | IC_50_^1^ vincristine (nM) | verapamil alone^2^ (% control) | IC_50_ vincristine (nM) |
| UKF-NB-3 | 0.25 ± 0.06 | 88 ± 10 | 0.22 ± 0.02 |
| UKF-NB-3^r^SNS-032^300nM^ | 2.8 ± 0.7  (10.8)^3^ | 92 ± 15 | 0.34 ± 0.12  (1.3)^4^ |
| UKF-NB-3^r^DOX^20^ | 23 ± 2  (90.5) | 98 ± 13 | 0.55 ± 0.10  (2.1) |
| UKF-NB-3^r^ETO^100^ | 0.92 ± 0.22  (3.6) | 94 ± 7 | 0.25 ± 0.05  (1.0) |
| UKF-NB-3^r^VCR^10^ | 105 ± 11  (414.3) | 97 ± 11 | 1.2 ± 0.2  (4.8) |

^1^ Concentration that inhibits cell viability by 50% as determined by MTT assay after 120h of incubation. Values are presented as mean ± S.D.

^2^ Effects of verapamil 10µM on cell viability in the absence of anti-cancer drugs as determined by MTT assay after 120h of incubation. Values are presented as mean ± S.D. relative to non-treated control cells.

^3^ IC_50_ resistant UKF-NB-3 sub-line/ IC_50_ UKF-NB-3

^4^ IC_50_ resistant UKF-NB-3 sub-line in the presence of verapamil/ IC_50_ UKF-NB-3

**Suppl. Table 1B**. Effects of siRNA-mediated ABCB1 depletion on SNS-032 sensitivity in UKF-NB-3 and UKF-NB-3^r^SNS-032^300nM^ cells.

|  | IC_50_^1^ SNS-032 (nM) | |
| --- | --- | --- |
|  | UKF-NB-3 | UKF-NB-3^r^SNS-032^300nM^ |
| no siRNA | 109 ± 26 | 720 ± 121 |
| non-targeting siRNA | 99 ± 37 | 811 ± 155 |
| ABCB1 siRNA | 89 ± 19 | 338 ± 6 |

^1^ Concentration that inhibits cell viability by 50% as determined by MTT assay after 120h of incubation. Values are presented as mean ± S.D.

**Suppl. Table 1C**. Effects of the ABCB1 inhibitor zosuquidar on the SNS-032 sensitivity of UKF-NB-3 and UKF-NB-3^r^SNS-032^300nM^ cells.

|  |  | + zosuquidar 2.5µM | |
| --- | --- | --- | --- |
| Cell line | IC_50_^1^ SNS-032 (nM) | zosuquidar alone^2^ (% control) | IC_50_ SNS-032 (nM) |
| UKF-NB-3 | 137.5 ± 11.7 | 102 ± 8 | 130.3 ± 4.3 |
| UKF-NB-3^r^SNS-032^300nM^ | 687.5 ± 31.4  (5.0)^3^ | 98 ± 10 | 151.5 ± 10.0  (1.1)^4^ |

^1^ Concentration that inhibits cell viability by 50% as determined by MTT assay after 120h of incubation. Values are presented as mean ± S.D.

^2^ Effects of zosuquidar 2.5µM on cell viability in the absence of anti-cancer drugs as determined by MTT assay after 120h of incubation. Values are presented as mean ± S.D. relative to non-treated control cells.

^3^ IC_50_ resistant UKF-NB-3 sub-line/ IC_50_ UKF-NB-3

^4^ IC_50_ resistant UKF-NB-3 sub-line in the presence of zosuquidar/ IC_50_ UKF-NB-3

**Suppl. Table 1D**. Effects of the non-ABCB1 substrate cisplatin on the viability of the neuroblastoma cell line UKF-NB-3 and its sub-lines with acquired resistance to SNS-032 (UKF-NB-3^r^SNS-032^300nM^), cisplatin (UKF-NB-3^r^CDDP^1000^), doxorubicin (UKF-NB-3^r^DOX^20^), etoposide (UKF-NB-3^r^ETO^100^), or vincristine (UKF-NB-3^r^VCR^10^).

| Cell line | IC_50_^1^ cisplatin (nM) |
| --- | --- |
| UKF-NB-3 | 280 ± 67 |
| UKF-NB-3^r^SNS-032^300nM^ | 373 ± 57  (1.3)^2^ |
| UKF-NB-3^r^CDDP^1000^ | 8936 ± 1377  (32.0) |
| UKF-NB-3^r^DOX^20^ | 710 ± 103  (2.5) |
| UKF-NB-3^r^ETO^100^ | 397 ± 93  (1.4) |
| UKF-NB-3^r^VCR^10^ | 1720 ± 327  (6.2) |

^1^ Concentration that inhibits cell viability by 50% as determined by MTT assay after 120h of incubation. Values are presented as mean ± S.D.

^2^ IC_50_ resistant UKF-NB-3 sub-line/ IC_50_ UKF-NB-3

**Suppl. Table 1E**. Effects of SNS-032, doxorubicin, etoposide, and vincristine on the viability of the neuroblastoma cell line UKF-NB-3 and its sub-line with acquired resistance to cisplatin (UKF-NB-3^r^CDDP^1000^).

| Cell line | IC_50_^1^ SNS-032 (nM) |
| --- | --- |
| UKF-NB-3 | 152.6 ± 32.8 |
| UKF-NB-3^r^CDDP^1000^ | 126.7 ± 28.0  (0.8)^2^ |
| Cell line | IC_50_^1^ doxorubicin (nM) |
| UKF-NB-3 | 9.0 ± 2.6 |
| UKF-NB-3^r^CDDP^1000^ | 36 ± 6  (4.0) |
| Cell line | IC_50_^1^ etoposide (nM) |
| UKF-NB-3 | 70 ± 5 |
| UKF-NB-3^r^CDDP^1000^ | 110 ± 17  (1.6) |
| Cell line | IC_50_^1^ vincristine (nM) |
| UKF-NB-3 | 0.25 ± 0.06 |
| UKF-NB-3^r^CDDP^1000^ | 0.84 ± 0.23  (3.3) |

^1^ Concentration that inhibits cell viability by 50% as determined by MTT assay after 120h of incubation. Values are presented as mean ± S.D.

^2^ IC_50_ resistant UKF-NB-3 sub-line/ IC_50_ UKF-NB-3

**Suppl. Table 1F**. Effects of the CDK2, 7, and 9 inhibitor seliciclib, the CDK9 inhibitor LDC000067, the CDK7 inhibitor BS-181, and the CDK 1,2,4,6,7, and 9 inhibitor alvocidib on the viability of the neuroblastoma cell line UKF-NB-3 and its sub-line with acquired resistance to SNS-032 (UKF-NB-3^r^SNS-032^300nM^).

| Cell line | IC_50_^1^ seliciclib (µM) |
| --- | --- |
| UKF-NB-3 | 22.3 ± 1.6 |
| UKF-NB-3^r^SNS-032^300nM^ | 31.6 ± 5.0  (1.4)^2^ |
| Cell line | IC_50_^1^ LDC000067 (µM) |
| UKF-NB-3 | 9.6 ± 1.5 |
| UKF-NB-3^r^SNS-032^300nM^ | 14.5 ± 1.4  (1.5)^2^ |
| Cell line | IC_50_^1^ BS-181 (µM) |
| UKF-NB-3 | 18.0 ± 4.5 |
| UKF-NB-3^r^SNS-032^300nM^ | 27.8 ± 1.7  (1.5)^2^ |

| Cell line | IC_50_^1^ alvocidib (nM) |
| --- | --- |
| UKF-NB-3 | 378 ± 18 |
| UKF-NB-3^r^SNS-032^300nM^ | 426 ± 33  (1.1)^2^ |

^1^ Concentration that inhibits cell viability by 50% as determined by MTT assay after 120h of incubation. Values are presented as mean ± S.D.

^2^ IC_50_ resistant UKF-NB-3 sub-line/ IC_50_ UKF-NB-3

**Suppl. Table 1G**. Effects of siRNA-mediated depletion of CDK7, CDK9, and CDK7 and CDK9 on the viability of the neuroblastoma cell line UKF-NB-3 and its sub-line with acquired resistance to SNS-032 (UKF-NB-3^r^SNS-032^300nM^) as determined by MTT assay 72h post-transfection.

| UKF-NB-3 | Viability  (% non-treated control) |
| --- | --- |
| non-targeting siRNA | 89.3 ± 3.1 |
| CDK7 siRNA | 88.7 ± 3.2 |
| CDK9 siRNA | 72.1 ± 4.1 |
| CDK7 siRNA + CDK9 siRNA | 44.5 ± 2.1 |
|  |  |
| UKF-NB-3^r^SNS-032^300nM^ | Viability  (% non-treated control) |
| non-targeting siRNA | 90.6 ± 4.9 |
| CDK7 siRNA | 85.8 ± 0.8 |
| CDK9 siRNA | 69.0 ± 0.3 |
| CDK7 siRNA + CDK9 siRNA | 52.2 ± 13.0 |

**Suppl. Table 1H**. Effects of the ABCB1 substrates SNS-032, doxorubicin, etoposide, and vincristine on the viability of the neuroblastoma cell line SHEP and its sub-line with acquired resistance to SNS-032 (SHEP^r^SNS-032^2000nM^).

|  |  | + verapamil 10µM | |
| --- | --- | --- | --- |
| Cell line | IC_50_^1^ SNS-032 (nM) | verapamil alone^2^ (% control) | IC_50_ SNS-032 (nM) |
| SHEP | 912.2 ± 18.7 | 102 ± 16 | 128.4 ± 31.1 |
| SHEP^r^SNS-032^2000nM^ | 5044.9 ± 421.5  (5.5)^3^ | 98 ± 11 | 254.0 ± 34.5  (2.0)^4^ |
|  |  |  |  |
|  |  | + verapamil 10µM | |
| Cell line | IC_50_^1^ doxorubicin (nM) | verapamil alone^2^ (% control) | IC_50_ doxorubicin (nM) |
| SHEP | 13.8 ± 0.7 | 102 ± 16 | 5.3 ± 1.1 |
| SHEP^r^SNS-032^2000nM^ | 68.8 ± 1.5  (5.0)^3^ | 98 ± 11 | 10.3 ± 0.4  (2.0)^4^ |

|  |  | + verapamil 10µM | |
| --- | --- | --- | --- |
| Cell line | IC_50_^1^ etoposide (nM) | verapamil alone^2^ (% control) | IC_50_ etoposide (nM) |
| SHEP | 194 ± 5 | 102 ± 16 | 93 ± 5 |
| SHEP^r^SNS-032^2000nM^ | 697 ± 112  (3.6)^3^ | 98 ± 11 | 270 ± 8  (2.9)^4^ |

|  |  |  |  |
| --- | --- | --- | --- |
|  |  | + verapamil 10µM | |
| Cell line | IC_50_^1^ vincristine (nM) | verapamil alone^2^ (% control) | IC_50_ vincristine (nM) |
| SHEP | 7.4 ± 0.1 | 102 ± 16 | 0.28 ± 0.04 |
| SHEP^r^SNS-032^2000nM^ | 36.4 ± 6.4  (4.9)^3^ | 98 ± 11 | 0.40 ± 0.06  (1.4)^4^ |

^1^ Concentration that inhibits cell viability by 50% as determined by MTT assay after 120h of incubation. Values are presented as mean ± S.D.

^2^ Effects of verapamil 10µM on cell viability in the absence of anti-cancer drugs as determined by MTT assay after 120h of incubation. Values are presented as mean ± S.D. relative to non-treated control cells.

^3^ IC_50_ SHEP^r^SNS-032^2000nM^ / IC_50_ SHEP

^4^ IC_50_ SHEP^r^SNS-032^2000nM^ in the presence of verapamil/ IC_50_ SHEP

**Suppl. Table 1I**. Effects of the non-ABCB1 substrate cisplatin, the CDK2, 7, and 9 inhibitor seliciclib, the CDK9 inhibitor LDC000067, the CDK7 inhibitor BS-181, and the CDK 1,2,4,6,7, and 9 inhibitor alvocidib on the viability of the neuroblastoma cell line SHEP and its sub-line with acquired resistance to SNS-032 (SHEP^r^SNS-032^2000nM^).

| Cell line | IC_50_^1^ cisplatin (nM) |
| --- | --- |
| SHEP | 583 ± 27 |
| SHEP^r^SNS-032^2000nM^ | 693 ± 37  (1.2)^2^ |
| Cell line | IC_50_^1^ seliciclib (µM) |
| SHEP | 21.2 ± 1.9 |
| SHEP^r^SNS-032^2000nM^ | 21.9 ± 0.9  (1.0)^2^ |
| Cell line | IC_50_^1^ LDC000067 (µM) |
| SHEP | 9.4 ± 0.8 |
| SHEP^r^SNS-032^2000nM^ | 12.1 ± 1.2  (1.3)^2^ |
| Cell line | IC_50_^1^ BS-181 (µM) |
| SHEP | 18.2 ± 1.1 |
| SHEP^r^SNS-032^2000nM^ | 28.8 ± 3.4  (1.6)^2^ |

| Cell line | IC_50_^1^ alvocidib (nM) |
| --- | --- |
| SHEP | 149 ± 42 |
| SHEP^r^SNS-032^2000nM^ | 146 ± 7  (1.0)^2^ |

^1^ Concentration that inhibits cell viability by 50% as determined by MTT assay after 120h of incubation. Values are presented as mean ± S.D.

^2^ IC_50_ SHEP^r^SNS-032^2000nM^ / IC_50_ SHEP

**Suppl. Table 1J**. Effects of SNS-032 or actinomycin D on the RNA polymerase activity in the neuroblastoma cell line SHEP and its sub-line with acquired resistance to SNS-032 (SHEP^r^SNS-032^2000nM^) in the absence or presence of the ABCB1 inhibitor verapamil (10µM) after 6h of incubation.

| SHEP |  |  |
| --- | --- | --- |
|  | RNA polymerase activity (% untreated control) | |
| Drug |  | + verapamil 10µM^1^ |
| SNS-032 100nM | 104 ± 1 | 78 ± 4 |
| SNS-032 300nM | 104 ± 3 | 38 ± 7 |
| SNS-032 600nM | 93 ± 3 | 22 ± 3 |
| SNS-032 1200nM | 70 ± 4 | 16 ± 3 |
| Actinomycin D 100ng/mL | 28 ± 3 | 19 ± 3 |
| SHEP^r^SNS-032^2000nM^ |  |  |
|  | RNA polymerase activity (% untreated control) | |
| Drug |  | + verapamil 10µM^1^ |
| SNS-032 100nM | 99 ± 3 | 101 ± 5 |
| SNS-032 300nM | 97 ± 3 | 56 ± 3 |
| SNS-032 600nM | 103 ± 2 | 32 ± 4 |
| SNS-032 1200nM | 99 ± 2 | 31 ± 5 |
| Actinomycin D 100ng/mL | 49 ± 3 | 19 ± 3 |

^1^ Please refer to Suppl. Table 1H for the effect of verapamil 10µM alone on SHEP or SHEP^r^SNS-032^2000nM^ cell viability

**Suppl. Table 1K**. Effects of the the CDK2, 7, and 9 inhibitor seliciclib, the CDK9 inhibitor LDC000067, the CDK7 inhibitor BS-181, and the CDK 1,2,4,6,7, and 9 inhibitor alvocidib on the viability of UKF-NB-3 sub-lines with acquired resistance to cytotoxic anti-cancer drugs.

| Cell line | IC_50_^1^ seliciclib (µM) |
| --- | --- |
| UKF-NB-3 | 22.3 ± 1.6 |
| UKF-NB-3^r^CDDP^1000^ | 17.2 ± 2.4  (0.8)^2^ |
| UKF-NB-3^r^DOX^20^ | 15.2 ± 2.5  (0.7)^2^ |
| UKF-NB-3^r^VCR^10^ | 24.6 ± 4.2  (1.1)^2^ |
|  |  |
| Cell line | IC_50_^1^ LDC000067 (µM) |
| UKF-NB-3 | 9.6 ± 1.5 |
| UKF-NB-3^r^CDDP^1000^ | 4.7 ± 0.3  (0.5)^2^ |
| UKF-NB-3^r^DOX^20^ | 8.9 ± 2.3  (0.9)^2^ |
| UKF-NB-3^r^VCR^10^ | 4.7 ± 1.6  (0.5)^2^ |
|  |  |
| Cell line | IC_50_^1^ BS-181 (µM) |
| UKF-NB-3 | 18.0 ± 4.5 |
| UKF-NB-3^r^CDDP^1000^ | 12.3 ± 4.0  (0.7)^2^ |
| UKF-NB-3^r^DOX^20^ | 9.4 ± 1.0  (0.5)^2^ |
| UKF-NB-3^r^VCR^10^ | 13.0 ± 0.7  (0.7)^2^ |

| Cell line | IC_50_^1^ alvocidib (nM) |
| --- | --- |
| UKF-NB-3 | 378 ± 18 |
| UKF-NB-3^r^CDDP^1000^ | 285 ± 41  (0.8)^2^ |
| UKF-NB-3^r^DOX^20^ | 238 ± 20  (0.6)^2^ |
| UKF-NB-3^r^VCR^10^ | 393 ± 17  (1.0)^2^ |

^1^ Concentration that inhibits cell viability by 50% as determined by MTT assay after 120h of incubation. Values are presented as mean ± S.D.

^2^ IC_50_ resistant UKF-NB-3 sub-line/ IC_50_ UKF-NB-3
